# Unexpected Inflammatory Effects of Intravaginal Gels (Universal Placebo Gel and Nonoxynol-9) on the Upper Female Reproductive Tract: A Randomized Crossover Study

**DOI:** 10.1371/journal.pone.0129769

**Published:** 2015-07-15

**Authors:** Karen Smith-McCune, Joseph C. Chen, Ruth M. Greenblatt, Uma Shanmugasundaram, Barbara L. Shacklett, Joan F. Hilton, Brittni Johnson, Juan C. Irwin, Linda C Giudice

**Affiliations:** 1 Department of Obstetrics, Gynecology and Reproductive Sciences, University of California San Francisco, San Francisco, California, United States of America; 2 Departments of Clinical Pharmacy and Internal Medicine, University of California San Francisco, San Francisco, California, United States of America; 3 Department of Epidemiology and Biostatistics, University of California San Francisco, San Francisco, California, United States of America; 4 Department of Medical Microbiology and Immunology, School of Medicine, University of California Davis, Davis, California, United States of America; University of Toronto, CANADA

## Abstract

Intravaginal anti-HIV microbicides could provide women with a self-controlled means for HIV prevention, but results from clinical trials have been largely disappointing. We postulated that unrecognized effects of intravaginal gels on the upper female reproductive tract might contribute to the lower-than-expected efficacy of HIV microbicides. Our objective was to study the effects of intravaginal gels on the immune microenvironment of the cervix and uterus. In this randomized crossover study, 27 healthy female volunteers used a nightly application of intravaginal nonoxynol-9 (N9) gel as a “failed” microbicide or the universal placebo gel (UPG) as a “safe” gel (intervention cycles), or nothing (control cycle) from the end of menses to the mid-luteal phase. At a specific time-point following ovulation, all participants underwent sample collection for measurements of T-cell phenotypes, gene expression, and cytokine/chemokine protein concentrations from 3 anatomic sites above the vagina: the cervical transformation zone, the endocervix and the endometrium. We used hierarchical statistical models to estimate mean (95% CI) intervention effects, for N9 and UPG relative to control. Exposure to N9 gel and UPG generated a common “harm signal” that included transcriptional up-regulation of inflammatory genes chemokine (C-C motif) ligand 20 (macrophage inflammatory factor-3alpha) and interleukin 8 in the cervix, decreased protein concentrations of secretory leukocyte protease inhibitor, and transcriptional up-regulation of inflammatory mediators glycodelin-A and osteopontin in the endometrium. These results need to be replicated with a larger sample, but underscore the need to consider the effects of microbicide agents and gel excipients on the upper female reproductive tract in studies of vaginal microbicides.

## Introduction

Vaginal microbicides are topical antimicrobials that block, kill, or inactivate HIV and/or other sexually transmitted pathogens when placed in the vagina prior to exposure. Wide deployment of safe and efficacious vaginal microbicides could provide women with a self-controlled means for protection from HIV, which in turn could reduce sexual transmission of HIV and impact the ongoing global HIV epidemic. Results from recent clinical trials of vaginal microbicides have been largely disappointing [1]. These results prompted a reappraisal of methods for selecting candidates for Phase III trials, including *in vitro* assays of efficacy and assessments of safety [1,2].

Nonoxynol-9 (N9), a nonionic detergent, was the first microbicide to be tested in large clinical trials for prevention of HIV transmission. Paradoxically, 3.5% N9 in a carbomer gel *increased* HIV transmission compared to carbomer gel alone in a high-risk population of women [3]. This was attributed in part to findings that N9 also increased risk of sexually transmitted infections and genital ulcers, presumably providing a portal for HIV access to target T-cells [4–6]. Subsequent investigations revealed that N9 injures epithelial cell membranes, resulting in the release of pro-inflammatory cytokines, recruitment of inflammatory mediators and efflux of macrophages in the vagina [7]. These findings prompted an increased focus on the safety of candidate microbicides with measurements of vaginal epithelial integrity and inflammatory infiltrates [8].

A recent approach for microbicide development has been to add antiretroviral drugs to vaginal gel formulations. Encouraging results from one trial of 1% tenofovir gel showed 39% protection against HIV infection [9], although in another study the use of tenofovir gel was terminated early due to lack of evidence of efficacy [10]. In the former trial, the effect of tenofovir gel was compared to the universal placebo gel (UPG), an agent used as a placebo control in many microbicide trials and assumed to be safe in the lower FRT [11].

Differences in the epithelial surfaces of the upper and lower FRT are likely to result in differences in their susceptibility to HIV infection [12]. While the vagina and ectocervix of the lower FRT are lined with a multi-layered squamous epithelium that is relatively resistant to injury, the upper FRT (including the endocervix and endometrium) is lined by a single layer of columnar epithelium that is a less effective barrier with closer proximity to underlying immune cells. The junction of the squamous and columnar cell types occurs on the cervix at the transformation zone (TZ). Fluctuations in sex hormones due to ovulation have been shown to suppress innate, adaptive and humoral immunity under the influence of progesterone (i.e., in the luteal phase of the menstrual cycle), leading to the concept of a “window of vulnerability” for HIV infection in the FRT and highlighting the need to consider the upper FRT in studies of HIV transmission [13,14]. We designed a study to test the hypothesis that intravaginal microbicides can gain access to and perturb the upper FRT, and thus could increase susceptibility of the upper FRT despite the intended effect of protecting the lower FRT. We studied the *in vivo* effects of 2 agents: N9 (an agent with known harmful effects on the lower FRT) and UPG (an agent used as a placebo control in many microbicide trials), compared to a no-treatment control, measuring biological effects on 3 anatomic sites above the vagina: the cervical TZ, the endocervix and the endometrium.

## Materials and Methods

### Overall study design

#### Ethics statement

The study protocol and consent materials were approved by the Committee on Human Research (approval # 10–01173), which is the ethics committee/institutional review board at University of California San Francisco. Signed informed consent was obtained from each participant.

#### Study design

This is a randomized 3-arm crossover study of the effects of 2 intravaginal products relative to a control on the upper FRT in healthy volunteers, with sample collection timed to be in the mid-luteal phase of the ovulatory cycle following 7–11 days’ exposure. Up to 3 time-periods (distinct ovulatory cycles) were studied per subject (see Table 1). This design reduces bias by controlling for personal characteristics that might affect outcomes, ensures balanced sample sizes across arms, and increases power to detect significant effects relative to a parallel-arm design. During the no-gel control cycle, participants placed no study product or applicator in the vagina. The study protocol, recruiting and consent materials were approved by the UCSF Committee on Human Research.

**Table 1:** Distribution of study participants and specimens analyzed, by study arm, anatomical site, and type of analysis. For a detailed breakdown of the numbers of specimens per exposure arm used for each analysis, see S1 Table.

Study arm	All	TZ*	Endocervix		Endometrium	
		Transcriptional	Immunologic	Protein	Immunologic	Transcriptional
N9	70 (35%)	20	17	12	12	9
UPG	63 (31%)	18	15	11	10	9
No gel	69 (34%)	18	16	12	14	9
All specimens	202	56	48	35	36	27
Participants:	27	27	25	19	21	17
Specimens per participant:	7.5	2.1	1.9	1.8	1.7	1.6

* TZ = cervical transformation zone

#### Recruitment of human volunteers

Healthy women volunteers were recruited via flyers placed in a variety of venues and advertisements in local publications. Exclusion factors were: positive HIV serology, positive urine nucleic acid amplification test for *Neisseria gonorrheae* or *Chlamydia trachomatis*, current or recent pregnancy or breastfeeding, age <18 or >44 years, recent history of irregular menstrual cycles and/or use of hormonal contraceptives, IUDs or vaginal products. Also excluded were volunteers using systemic corticosteroids or immune modulating therapies, and women unwilling/unable to refrain from vaginal intercourse for at least 72 hours before specimen collection or to use non-lubricated condoms during study cycles. Signed informed consent was obtained from each participant. Women received payment to compensate for time and effort required for each study visit.

#### Clinical study procedures

After an initial screening and enrollment visit, participants were instructed in study procedures as follows. Participants were asked to use vaginal products (Conceptrol N9 gel or UPG) or no product during an ovulatory cycle and to return after each exposure period, for collection of clinical samples to assess treatment effects. Conceptrol (Revive Personal Products, Madison, New Jersey) contains 4% N9 in a gel with lactic acid, methylparaben, povidone, propylene glycol, purified water, sodium carboxymethyl cellulose, sorbic acid, and sorbitol solution. UPG (CONRAD, Arlington, VA) is an isotonic non-buffering gel containing 2.7% hydroxyethylcellulose, sorbic acid, sodium hydroxide, sodium chloride and purified water. Cycles in which participants used products were followed by “washout” menstrual cycles during which no study intervention or biological sampling occurred. Early in the study, volunteers were randomized to N9 then UPG or the reverse sequence. The no-gel (and no-applicator) cycle was introduced to the study protocol part-way through the study, and women who had participated prior to this modification were invited back for a third study cycle. The sequence of three study conditions was randomly assigned when a participant met eligibility criteria via telephone screening and was scheduled for a visit.

Beginning after cessation of menstrual bleeding, consenting participants were instructed to use the assigned intravaginal product every night until 7–11 days after the luteinizing hormone (LH) surge was identified via a urine home detection kit (Clearblue(r) Ovulation Test DIGITAL, Proctor & Gamble, Cincinnati, OH). Within 7–11 days of urine LH detection (i.e., after ovulation), a study visit occurred for collection of study specimens. Prior to specimen collection, participants were asked if they had engaged in vaginal intercourse within the prior 72 hours, and if they had, the visit was canceled and the participants were asked to repeat the intervention during a subsequent menstrual cycle. Peripheral blood was collected in EDTA tubes. A speculum was inserted into the vagina, the cervix was visualized, and the following specimens were collected: endocervical fluid via wick (an ophthalmic sponge [Merocel eye spears, Beaver Visitec International, Waltham, MA] inserted into the canal for 90 sec, followed by a second identical collection); endocervical curettage (mechanical scraping) sample using a Kevorkian curette with basket square tip; a cervical biopsy (performed at the transformation zone if visible or at the os using a Mini-Tischler punch biopsy forceps); and an endometrial biopsy using with a 3 mm cannula (Miltex brand Softflex) inserted through the internal os into the endometrial cavity. For the latter procedure, if insertion of the cannula was difficult, local anesthesia was provided via cervical injection with lidocaine, and then a tenaculum was applied to the ectocervix. A second pass with the curette was made if the amount of tissue was observed to be insufficient after the first pass.

The occurrence of ovulation was confirmed by a serum progesterone level >2 ng/ml and by endometrial histology [15]. The mean progesterone in the overall sample was 9.27 ng/ml (95% CI 7.94–10.6).

#### Statistical Methods

Participants were invited to complete three study arms, but dropouts occurred due to difficulties with scheduling and unwillingness to undergo repeated specimen collection. We report the number of volunteers who contributed post-exposure specimens and the number of specimens analyzed, by study arm, anatomical site, and sampling technique (cervical biopsy, endocervical wick, endocervical curettage, and endometrial biopsy) (Table 1 and S1 Table).

### Gene expression profiling of cervical and endometrial samples

#### Microarray Analysis

Cervical and endometrial biopsies were snap frozen at the time of collection and stored at -80°C until use. RNA was isolated using the Nucleospin RNA purification kit (Machery Nagel, Bethlehem, PA) following the manufacturer’s protocol including DNase treatment. The purity and integrity of all RNA samples were confirmed through Nanodrop (Nanodrop, Wilmington, DE) and Bioanalyzer 255 (Agilent, Santa Clara, CA), respectively. Samples that did not meet the standards required for microarray analysis (e.g. due to ribosomal RNA degradation or phenol/guanidine salt contamination) were excluded. Some samples also were found to consist of mucous and/or blood, not tissue, after resuspension in Trizol. These samples were considered non-representative of the tissue of origin and were also excluded from microarray analysis. Samples were sent to the UCSF/Gladstone Institutes Genomics Core for cDNA synthesis and hybridization to Human Gene 1.0 ST arrays (Affymetrix, Santa Clara, CA), with updated annotations, probing 36,079 transcripts and 21,014 genes, as previously reported [16]. Briefly, the intensity values of different probe sets (genes) in the GeneChip Operating Software (Affymetrix) were imported into GeneSpring GX 11.02 software (Agilent Technologies, Santa Clara, CA) and processed using the robust multiarray analysis algorithm for background adjustment, normalization, and log2 transformation of perfect match values. RMA16 was utilized as the background correction algorithm for ST array technology. The CEL files obtained from array studies are available in the Gene Expression Omnibus (GEO) at the National Center for Bioinformatic Information (NCBI).

#### Microarray Validation with Quantitative Real-Time PCR (qPCR)

To validate a subset of differentially expressed genes identified in the microarray analysis, qPCR was used as previously described [17]. Briefly, 10ng of total cDNA, obtained from post-array amplification of mRNA were combined with the Maxima Syber Green reagent (K0222, Thermo Scientific, Rockford, IL) and 300nM of pooled custom-designed forward and reverse primers (Fluidigm, South San Francisco, CA) and analyzed using the Strategene MX3005 system (Agilent, Santa Clara, CA). The efficiency of each primer was calculated through serial dilution of sample cDNA, and all primers expressed 90–100% efficiency. HPRT1 was utilized as a housekeeping gene due to its stability of expression between treatment arms. Absence of non-specific amplification and primer dimers was confirmed through the dissociation curve, and the comparative Ct method (delta delta Ct) was utilized to calculate the relative expression levels of each target gene for each treatment (ABI User Bulletin 2). Fold changes were calculated through treatment (N9 or UPG) normalized to the no-gel arm.

#### Statistical methods

Differential expression analysis of microarray data, to estimate fold-changes in cervical and endometrial biopsy tissue from N9 or UPG intervention versus control (no-gel) was conducted by the Giudice lab using Genespring 12.1 (2013). We report genes with ≥1.5-fold changes and P<0.05 by two-way ANOVA with Benjamini-Hochberg multiple-testing correction for false discovery rate. Quantitative RT-PCR data was analyzed by t-tests to determine significant differences in gene expression in each comparison arm using R-Commander (2011) and Microsoft Excel (2010). Ingenuity Pathway Analysis (Ingenuity Systems Inc, Redwood City, CA) was used to evaluate the molecular, cellular, and physiological functional pathways that were affected by the differentially expressed genes in both the endometrium and cervix after N9 or UPG exposure compared to no-gel. Resulting pathways utilized for analysis were derived from genes with a ≥1.5 fold change.

### Phenotypic and functional assays of cervical and endometrial T-cells

#### Mononuclear Cell Isolation

Samples of blood, endocervical curettage and endometrial biopsies collected at UCSF were transported on ice to UC Davis to arrive within 3–4 hours of collection. Upon arrival, peripheral blood mononuclear cells (PBMC) were isolated by Ficoll-Hypaque (Pfizer, New York, NY) density gradient centrifugation, and washed in PBS. Mononuclear cells from endocervical curettage were isolated by repeated pipetting, followed by passage through a 70μm nylon cell strainer (Becton Dickinson, Bedford, MA) and washing in complete medium [RPMI-1640 supplemented with 15% FBS, penicillin (100U/ml), streptomycin (100μg/ml), and glutamine (2mM)]. Endometrial biopsies were subjected to two to three rounds of collagenase type II digestion (0.5 mg/ml; Sigma-Aldrich, St. Louis, MO), followed by mechanical disruption using an 18-gauge blunt-end needle and passage through a 70-μm nylon cell strainer. The pooled cells were washed in complete medium.

Red blood cell lysis was performed as needed on PBMC, endometrium and endocervical cells using ammonium chloride-potassium carbonate-EDTA (ACK).

#### Monoclonal Antibodies

Fluorochrome-labeled monoclonal antibodies used for the phenotypic and intracellular staining assay included CD3 (clone UCHT1), CD4 (clone RPA-T4), CD8 (clone SK1), CCR7 (clone 3D12), CXCR4 (clone12G5), CCR5 (clone 2D7), CD8 (clone RPA-T8), IFNγ (clone B27), TNFα (clone MAb11), MIP-1ß (clone D21-1351) from Becton-Dickinson Pharmingen (San Diego, CA); CD45RA (2H4), CD4 (clone T4D11) from Beckman Coulter (Fullerton, CA); CD38 (clone HB7), CD107a (clone H4A3), and IL10 (clone JES3-19F1) from BD Biosciences (San Jose, CA); HLADR (clone TU36), aqua amine reactive dye from Invitrogen (Carlsbad, CA); CD66b (G10F5) Biolegend (San Diego, CA); and IL17 (clone eBio64CAP17), IL2 (clone MQ1-17H12), from eBioscience (San Diego, CA). Optimum antibody titers were determined empirically for each antibody based on preliminary titration experiments using serial dilutions, which included the manufacturers’ recommended amounts. Flow cytometry data were acquired on an LSRII (BD Immunocytometry Systems, San Jose, CA) equipped with 405, 488, and 643 nm lasers and utilizing FACSDIVA software (BDIS). Analysis of cytometry data was done with FlowJo software (TreeStar, Ashland, OR). Results were recorded as the percentage of CD4+ or CD8+ T-cells expressing a given surface marker or combination of markers.

#### Statistical Methods

Preliminary histograms of T-cell phenotypes measured at two anatomical sites (endocervix via curettage, and endometrium via biopsy) revealed that distributions were typically right-skewed; consequently, most analyses were conducted on log-transformed outcomes and the remainder were conducted on the natural scale. Separately for CD4+ T-cells (83 values) and CD8+ T-cells (81 values), we used random-effects regression to model outcomes as a function of intervention arm, anatomical site, and the arm-by-site interaction. We report estimates on the natural scale of within-site mean (95% CI) frequencies following control (no-gel) cycles, after transforming geometric means to arithmetic means. We also report mean (95% CI) intervention vs. no-gel effects and corresponding chi-squared tests. The fit of each model was judged via analyses of residuals, including Kolmogorov-Smirnov tests. Models that fit poorly due to a small number of outliers were reanalyzed in 5% (or 10%)-Winsorized samples. Of 16 CD4+ phenotypes, 13 were analyzed on the log-scale (intervention effects are estimated as fold-changes), and 2 on the natural scale (intervention effects are estimated as differences). Of 16 CD8+ phenotypes, 12 were analyzed on the log-scale and 4 on the natural scale. Companion nonparametric analyses were not performed because the paired samples would be quite small within T-cell type, anatomical site, and intervention arm. All analyses were conducted using SAS version 9.4.

### Endocervical wick cytokine measurements

Endocervical wick samples were snap frozen at the time of collection and stored at -80°C until analysis. Wick samples were weighed and then extracted following published techniques into 300ul ice cold extraction buffer (PBS, 0.25M NaCl + 0.1mg/ml aprotinin), centrifuged, and extracted a second time in 300 ul of extraction buffer [18]. Wicks were allowed to air dry for 24 hours then weighed again; the dry wick weight was subtracted from the initial wet weight to determine the volume of fluid extracted from each wick. The mean [wet wick weight—dry wick weight] was 60.1 mg (range 0.9–378.3). Two wick samples were collected consecutively from each participant and the 2 samples were pooled for each participant for analysis. Samples were assayed for the following cytokines on the Milliplex panel (Millipore, Billerica, MA): IFNα2, IFNγ, IL10, IL12p70, IL1α, IL1β, IL6, IL8, MCP1, MIP1α, MIP1ß, RANTES and TNFα. The plates were read on a Bio-Plex Suspension Array Reader (Bio-Rad Laboratories Inc, CA, USA). Samples were also assayed using commercial ELISA kits for human neutrophil peptides 1–3 (HNP1-3) (Hycult Biotech, Plymouth Meeting, PA), lactoferrin (Novus Biologicals, Littleton, CO) and secretory leukocyte protease inhibitor (SLPI) (R&D Systems, Minneapolis, MN). Spike experiments have excluded interference of N9 or UPG with the Luminex or ELISA assays at 5% gel concentrations, which approximates the expected dilution of the gel into the 600 μl extraction buffer. Higher concentrations of the gels may have interfered with the Luminex assay due to high viscosity. Protein concentrations measured in the assays were multiplied by the dilution factor ([wet wick weight—dry wick weight] + 600 divided by [wet wick weight—dry wick weight]) to calculate the concentration (pg/ml) of each factor in the wick fluid. Fluid extracted from the wicks had to be further diluted for the ELISA assays in order to obtain readings within the range of the standard curve, as follows: SLPI 1/1300, lactoferrin 1/3000, HNP1-3 1/250. Samples with a [wet wick weight—dry wick weight] of less than 2 mg were excluded from the analysis as this small amount of fluid was not felt to be representative of the endocervical environment.

#### Statistical analysis

Preliminary histograms revealed that distributions of protein levels measured in cervical wicks were right-skewed; consequently all analyses were conducted on log-transformed outcomes. Initial comparisons of within-person paired outcomes (N9 versus no-gel, and UPG versus no-gel) were based on Wilcoxon Signed-Rank (WSR) tests of no difference between distributions. We also used a parametric approach in order to estimate mean (95% CI) protein concentrations following control (no-gel) cycles and fold-changes between intervention and control outcomes, and conduct chi-squared tests. Because of the small number of outcomes, we Winsorized (5%) the log-transformed outcomes prior to analysis. For biomarkers with 56 values, 5% Winsorization typically replaced the two lowest and two highest outcomes with the values at the 5^th^ and 95^th^ percentiles of the distribution, respectively; however, if an extreme value occurred in multiple subjects/arms, the imputation was applied to each. We used mixed-effects regression to model the transformed outcomes as a function of intervention arm, accounting for multiple outcomes per subject via random effects. The fit of a model was judged via analyses of residuals, including Kolmogorov-Smirnov tests. Results, reported on the natural scale, include (arithmetic) mean protein concentrations in the untreated arm and intervention effects relative to no-gel, expressed as mean (95% CI) levels and fold-changes, respectively.

## Results

### Participant recruitment, visit completion and specimen collection

The overall study population was 28% black, 44% white, 13% Asian and 15% other/mixed. The mean age of participants was 35.1 years (range 24–43, 95% CI 34.0–36.2). The mean day of the menstrual cycle at the time of the biopsy visit was 22.7 (95% CI 21.7–24.1). The mean numbers of days post LH-surge was 8.3 (95% CI 7.9–8.8). The mean number of days of product exposure was 17.3 (95% CI, 13.2–21.4). None of these features varied by arm.

While participants were asked to complete all 3 study arms, some women did not, due to difficulties with scheduling and unwillingness to undergo repeated biopsies. Of 27 volunteers, 12 contributed specimens for all 3 exposure cycles, 7 for 2 cycles, and 8 for a single cycle (Table 1). On average, 1.6 to 2.1 exposures per woman were available for analysis (Table 1). By arm, the number of participants contributing specimens and the number of specimens contributed were approximately equal, overall and by site/method; however, specimens were more often contributed when the procedures to collect the specimens were less invasive (e.g. more wick samples were contributed than endometrial biopsies).

### Effects of N9 and UPG on the transcriptome of the cervical transformation zone (TZ)

In cervical TZ samples (35 samples from 21 participants), N9 exposure resulted in up (33 genes)-or down (86 genes)-regulation (≥1.5 fold, p<0.05) of 119 genes compared to no-gel samples. The complete list of differentially expressed genes is presented in S2 Table. A partial list of genes with altered expression that were validated by PCR is presented in Table 2. N9 resulted in up-regulation of genes associated with inflammation and chemotaxis including CCL2, CCL19, CCL20, IL1α, IL1β, IL6, and IL8 [19–24]. Pathways associated with cell-to-cell signaling, cellular movement and inflammatory response were altered and reached statistical significance (Z score >2.0) in Ingenuity Pathway Analysis (Table 3). Many of the functions that were altered would be expected to result in increases in chemotaxis, movement and adhesion of potential HIV target cells including lymphocytes, monocytes and phagocytes.

**Table 2:** Selected list of differentially expressed genes in cervical and endometrial biopsy samples from women using N9 or UPG versus no-gel measured by array (minimum 1.5 fold change, p<0.05) and validated by qRT-PCR. Genes are arranged from most highly up-regulated at the top to most highly down-regulated at the bottom based on results from the array data. Genes whose expression patterns are similarly altered by N9 and UPG are highlighted with bold text.

Cervix Transformation Zone: N9 vs No Gel	Gene symbol	Fold Change*	
Gene description		array	qRT-PCR
**interleukin 8**	**IL8**	2.18 Up	2.95 Up
amphiregulin	AREG	2.18 Up	15.8 Up
**chemokine (C-C motif) ligand 20**	**CCL20**	2.12 Up	3.91 Up
interleukin 1, alpha	IL1A	1.91 Up	5.97 Up
selectin E	SELE	1.87 Up	1.99 Up
interleukin 1, beta	IL1B	1.82 Up	1.84 Up
chemokine (C-C motif) ligand 19	CCL19	1.80 Up	4.74 Up
chemokine (C-X-C motif) ligand 2	CXCL2	1.64 Up	6.04 Up
interleukin 6 (interferon, beta 2)	IL6	1.57 Up	1.83 Up
chemokine (C-C motif) ligand 2	CCL2	1.51 Up	2.34 Up
**serpin peptidase inhibitor, clade B (ovalbumin), member 12**	**SERPINB12**	2.49 Down	2.16 Down
serine peptidase inhibitor, Kazal type 7 (putative)	SPINK7	2.65 Down	2.05 Down
**keratin 1**	**KRT1**	3.06 Down	21.3 Down
**Cervix Transformation Zone: UPG vs No Gel**			
**chemokine (C-C motif) ligand 20**	**CCL20**	1.89 Up	3.69 Up
**interleukin 8**	**IL8**	1.52 Up	2.89 Up
chemokine (C-X-C motif) ligand 5	CXCL5	1.50 Up	1.54 Up
**serpin peptidase inhibitor, clade B (ovalbumin), member 12**	**SERPINB12**	1.66 Down	1.71 Down
keratin 10	KRT10	1.96 Down	NPA^§^
**keratin 1**	**KRT1**	2.05 Down	3.72 Down
**Endometrium: N9 vs No Gel**			
phospholipase A2, group IIA (platelets, synovial fluid)	PLA2G2A	2.06 Up	33.2 Up
**progestagen-associated endometrial protein**	**PAEP**	1.95 Up	1.76 Up
**secreted phosphoprotein 1**	**SPP1**	1.65 Up	4.58 Up
**killer cell immunoglobulin-like receptor family proteins** ^**¶**^	**KIRs**	1.54 Up	NPA^§^
fibronectin 1	FN1	1.52 Down	7.71 Down
**matrix metallopeptidase 26**	**MMP26**	1.71 Down	5.17 Down
**serpin peptidase inhibitor, clade A, member 5**	SERPINA5	2.06 Down	3.50 Down
**matrix metallopeptidase 7 (matrilysin, uterine)**	**MMP7**	2.11 Down	3.61 Down
**Endometrium: UPG vs No Gel**			
**progestagen-associated endometrial protein**	**PAEP**	4.07 Up	11.6 Up
**secreted phosphoprotein 1**	**SPP1**	2.58 Up	4.68 Up
complement component 3	C3	2.22 Up	2.54 Up
serpin peptidase inhibitor, clade G (C1 inhibitor), member 1	SERPING1	2.16 Up	2.87 Up
chemokine (C-X-C motif) ligand 13	CXCL13	1.70 Up	1.64 Up
interleukin 15	IL15	1.66 Up	1.50 Up
chemokine (C-C motif) ligand 21	CCL21	1.56 Up	1.92 Up
**killer cell immunoglobulin-like receptor family proteins** ^**¶**^	**KIRs**	1.52 Up	NPA^§^
**matrix metallopeptidase 7 (matrilysin, uterine)**	**MMP7**	1.70 Down	1.84 Down
serpin peptidase inhibitor, clade B (ovalbumin), member 9	SERPINB9	1.99 Down	3.10 Down
**serpin peptidase inhibitor, clade A, member 5**	SERPINA5	3.28 Down	10.3 Down
**matrix metallopeptidase 26**	**MMP26**	4.82 Down	2.02 Down

Samples analyzed: (i) Cervix: No gel, 16; N9, 13; UPG, 11 (ii) Endometrium: No gel, 11; N9, 10; UPG, 10

* Up and Down refers to up- or down-regulated expression relative to No Gel

^¶^ KIR3DS1|KIR3DL1|KIR2DS1|KIR2DL3|KIR2

^§^ NPA no primer available

**Table 3:** Top biofunctions affected by differential gene expression in cervix biopsy samples from women using N9 compared to no gel. Biofunction analysis conducted through Ingenuity Pathway Analysis (IPA), Z scores ≥1.5. Pathways present in more than one category are represented only once.

Category	Disease or Functions	Predicted Activity	Z-score	Molecules	#*
Immune Cell Trafficking	adhesion of immune cells	Increased	2.74	CCL19,CCL2,CCL20,IL1A,IL1B,IL6,IL8,SELE	8
	adhesion of mononuclear leukocytes	Increased	2.43	CCL19,CCL2,CCL20,IL1A,IL6,SELE	6
	adhesion of lymphocytes	Increased	2.24	CCL19,CCL2,CCL20,IL6,SELE	5
	activation of phagocytes	Increased	2.20	CCL2,IL1A,IL1B,IL6,IL8	5
	chemotaxis of mononuclear leukocytes	Increased	2.19	CCL19,CCL2,CCL20,IL1B,IL8,SELE	6
	cell movement of myeloid cells	Increased	2.11	CCL2,CCL20,CXCL2,IL1A,IL1B,IL6,IL8,SELE	8
	cell movement of phagocytes	Increased	2.09	CCL19,CCL2,CCL20,CXCL2,IL1A,IL1B,IL6,IL8,SELE	9
	chemotaxis of phagocytes	Increased	2.02	CCL19,CCL2,CCL20,CXCL2,IL1B,IL8,SELE	7
	chemotaxis of T lymphocytes	Increased	2.00	CCL19,CCL2,CCL20,IL8	4
	chemoattraction of phagocytes	Increased	1.98	CCL19,CCL2,CCL20,IL8	4
	adhesion of phagocytes	Increased	1.97	IL1A,IL1B,IL8,SELE	4
	activation of myeloid cells	Increased	1.96	CCL2,IL1A,IL1B,IL8	4
	cell movement of PBMCs	Increased	1.96	CCL19,CCL2,CCL20,IL8	4
	cell movement of leukocytes	Increased	1.95	CCL19,CCL2,CCL20,CXCL2,IL18,IL1A,IL1B,IL6,IL8,SELE	10
	cell movement of monocytes	Increased	1.94	CCL2,CCL20,IL1B,IL8,SELE	5
	cell movement of mononuclear leukocytes	Increased	1.91	CCL19,CCL2,CCL20,IL18,IL1B,IL8,SELE	7
	lymphocyte migration	Increased	1.85	CCL19,CCL2,CCL20,IL18,IL8,SELE	6
	chemotaxis of myeloid cells	Increased	1.67	CCL2,CXCL2,IL1B,IL8,SELE	5
	migration of phagocytes	Increased	1.67	CCL19,CCL2,CCL20,IL1B,IL6,IL8,SELE	7
	activation of leukocytes	Increased	1.55	CCL2,CEACAM5,IL1A,IL1B,IL6,IL8	6
Inflammatory Response	inflammatory response	Increased	2.62	CCL19,CCL2,CCL20,CXCL2,IL1A,IL1B,IL6,IL8,SELE	9
	adhesion of endothelial cells	Increased	2.22	CCL2,IL1A,IL1B,IL8,SELE	5
Cell-To-Cell Signaling and Interaction	chemoattraction of cells	Increased	2.21	CCL19,CCL2,CCL20,IL1B,IL8	5
	binding of leukocytes	Increased	2.16	CCL19,CCL2,CCL20,IL8,SELE	5
	adhesion of vascular endothelial cells	Increased	2.00	CCL2,IL1A,IL8,SELE	4
	adhesion of kidney cells	Increased	1.97	CCL2,HBEGF,IL8,KLK6	4
	binding of cells	Increased	1.94	AREG/AREGB,CCL19,CCL2,CCL20,IL1B,IL6,IL8,KRT1,SELE,TNFRSF12A	10
	activation of cells	Increased	1.55	AREG/AREGB,CCL2,CEACAM5,HBEGF,IL1A,IL1B,IL6,IL8	8
Cellular Development	proliferation of tumor cell lines	Increased	2.48	AREG/AREGB,CEACAM6,HBEGF,IL1A,IL1B,IL6,IL8,KRT10,SLURP1,SPINK7	10
	proliferation of smooth muscle cells	Increased	2.41	CCL2,HBEGF,IL1A,IL1B,IL6,IL8	6
	differentiation of leukocytes	Increased	2.16	CCL2,IL1A,IL1B,IL6,IL8,SPINK5	6
	differentiation of mononuclear leukocytes	Increased	1.92	CCL2,IL1B,IL6,IL8,SPINK5	5
	differentiation of blood cells	Increased	1.64	CCL2,IL1A,IL1B,IL6,IL8,SPINK5,TNFRSF12A	7
	differentiation of cells	Increased	1.60	AREG/AREGB,CCL2,HBEGF,IL1A,IL1B,IL6,IL8,MAL,SCEL,SPINK5,TNFRSF12A,UPK1A	12
	cell movement	Increased	2.75	AREG/AREGB,CCL19,CCL2,CCL20,CHI3L1,CLU,CXCL2,FAP,HBEGF,IL18,IL1A,IL1B,IL6,IL8,SELE,TNFRSF12A	16
	chemotaxis of cells	Increased	2.55	CCL19,CCL2,CCL20,CXCL2,HBEGF,IL1B,IL8,SELE	8
Cellular Movement	migration of cells	Increased	2.48	AREG/AREGB,CCL19,CCL2,CCL20,CHI3L1,CXCL2,FAP,HBEGF,IL18,IL1A,IL1B,IL6,IL8,SELE,TNFRSF12A	15
	invasion of cells	Increased	2.36	AREG/AREGB,CCL19,CCL2,CHI3L1,CLU,HBEGF,IER3,IL18,IL8	9
	chemoattraction of cells	Increased	2.21	CCL19,CCL2,CCL20,IL1B,IL8	5
Hematological System Development and Function	quantity of blood cells	Increased	1.67	CCL2,IL1A,IL1B,IL6,IL8	5
	differentiation of blood cells	Increased	1.64	CCL2,IL1A,IL1B,IL6,IL8,SPINK5,TNFRSF12A	7
Skeletal and Muscular System Development and Function	proliferation of smooth muscle cells	Increased	2.41	CCL2,HBEGF,IL1A,IL1B,IL6,IL8	6
Immune Cell Trafficking	adhesion of immune cells	Increased	2.74	CCL19,CCL2,CCL20,IL1A,IL1B,IL6,IL8,SELE	8
	adhesion of mononuclear leukocytes	Increased	2.43	CCL19,CCL2,CCL20,IL1A,IL6,SELE	6
	adhesion of lymphocytes	Increased	2.24	CCL19,CCL2,CCL20,IL6,SELE	5
	activation of phagocytes	Increased	2.20	CCL2,IL1A,IL1B,IL6,IL8	5
	chemotaxis of mononuclear leukocytes	Increased	2.19	CCL19,CCL2,CCL20,IL1B,IL8,SELE	6
	cell movement of myeloid cells	Increased	2.11	CCL2,CCL20,CXCL2,IL1A,IL1B,IL6,IL8,SELE	8
	cell movement of phagocytes	Increased	2.09	CCL19,CCL2,CCL20,CXCL2,IL1A,IL1B,IL6,IL8,SELE	9
	chemotaxis of phagocytes	Increased	2.02	CCL19,CCL2,CCL20,CXCL2,IL1B,IL8,SELE	7
	chemotaxis of T lymphocytes	Increased	2.00	CCL19,CCL2,CCL20,IL8	4
	chemoattraction of phagocytes	Increased	1.98	CCL19,CCL2,CCL20,IL8	4
	adhesion of phagocytes	Increased	1.97	IL1A,IL1B,IL8,SELE	4
	activation of myeloid cells	Increased	1.96	CCL2,IL1A,IL1B,IL8	4
	cell movement of PBMCs	Increased	1.96	CCL19,CCL2,CCL20,IL8	4
	cell movement of leukocytes	Increased	1.95	CCL19,CCL2,CCL20,CXCL2,IL18,IL1A,IL1B,IL6,IL8,SELE	10
	cell movement of monocytes	Increased	1.94	CCL2,CCL20,IL1B,IL8,SELE	5
	cell movement of mononuclear leukocytes	Increased	1.91	CCL19,CCL2,CCL20,IL18,IL1B,IL8,SELE	7
	lymphocyte migration	Increased	1.85	CCL19,CCL2,CCL20,IL18,IL8,SELE	6
	chemotaxis of myeloid cells	Increased	1.67	CCL2,CXCL2,IL1B,IL8,SELE	5
	migration of phagocytes	Increased	1.67	CCL19,CCL2,CCL20,IL1B,IL6,IL8,SELE	7
	activation of leukocytes	Increased	1.55	CCL2,CEACAM5,IL1A,IL1B,IL6,IL8	6
Inflammatory Response	inflammatory response	Increased	2.62	CCL19,CCL2,CCL20,CXCL2,IL1A,IL1B,IL6,IL8,SELE	9
	adhesion of endothelial cells	Increased	2.22	CCL2,IL1A,IL1B,IL8,SELE	5
Cell-To-Cell Signaling and Interaction	chemoattraction of cells	Increased	2.21	CCL19,CCL2,CCL20,IL1B,IL8	5
	binding of leukocytes	Increased	2.16	CCL19,CCL2,CCL20,IL8,SELE	5
	adhesion of vascular endothelial cells	Increased	2.00	CCL2,IL1A,IL8,SELE	4
	adhesion of kidney cells	Increased	1.97	CCL2,HBEGF,IL8,KLK6	4
	binding of cells	Increased	1.94	AREG/AREGB,CCL19,CCL2,CCL20,IL1B,IL6,IL8,KRT1,SELE,TNFRSF12A	10
	activation of cells	Increased	1.55	AREG/AREGB,CCL2,CEACAM5,HBEGF,IL1A,IL1B,IL6,IL8	8
Cellular Development	proliferation of tumor cell lines	Increased	2.48	AREG/AREGB,CEACAM6,HBEGF,IL1A,IL1B,IL6,IL8,KRT10,SLURP1,SPINK7	10
	proliferation of smooth muscle cells	Increased	2.41	CCL2,HBEGF,IL1A,IL1B,IL6,IL8	6
	differentiation of leukocytes	Increased	2.16	CCL2,IL1A,IL1B,IL6,IL8,SPINK5	6
	differentiation of mononuclear leukocytes	Increased	1.92	CCL2,IL1B,IL6,IL8,SPINK5	5
	differentiation of blood cells	Increased	1.64	CCL2,IL1A,IL1B,IL6,IL8,SPINK5,TNFRSF12A	7
	differentiation of cells	Increased	1.60	AREG/AREGB,CCL2,HBEGF,IL1A,IL1B,IL6,IL8,MAL,SCEL,SPINK5,TNFRSF12A,UPK1A	12
	cell movement	Increased	2.75	AREG/AREGB,CCL19,CCL2,CCL20,CHI3L1,CLU,CXCL2,FAP,HBEGF,IL18,IL1A,IL1B,IL6,IL8,SELE,TNFRSF12A	16
	chemotaxis of cells	Increased	2.55	CCL19,CCL2,CCL20,CXCL2,HBEGF,IL1B,IL8,SELE	8
Cellular Movement	migration of cells	Increased	2.48	AREG/AREGB,CCL19,CCL2,CCL20,CHI3L1,CXCL2,FAP,HBEGF,IL18,IL1A,IL1B,IL6,IL8,SELE,TNFRSF12A	15
	invasion of cells	Increased	2.36	AREG/AREGB,CCL19,CCL2,CHI3L1,CLU,HBEGF,IER3,IL18,IL8	9
	chemoattraction of cells	Increased	2.21	CCL19,CCL2,CCL20,IL1B,IL8	5
Hematological System Development and Function	quantity of blood cells	Increased	1.67	CCL2,IL1A,IL1B,IL6,IL8	5
	differentiation of blood cells	Increased	1.64	CCL2,IL1A,IL1B,IL6,IL8,SPINK5,TNFRSF12A	7
Skeletal and Muscular System Development and Function	proliferation of smooth muscle cells	Increased	2.41	CCL2,HBEGF,IL1A,IL1B,IL6,IL8	6

* number of molecules

In cervical TZ samples, UPG exposure resulted in up (8 genes)-or down (6 genes)-regulation (≥1.5 fold, p<0.05) of 14 genes compared to no-gel samples. The complete list of differentially expressed genes is presented in S3 Table. Genes of interest with altered expression that were validated by PCR included several immune modulators such as CCL20 [20], IL8 [24] and CXCL5 [25] (Table 2). Pathway analysis did not indicate any significant effects of UPG on cervical TZ.

### Effects of N9 and UPG on protein expression in the endocervical canal

**Fig 1:**
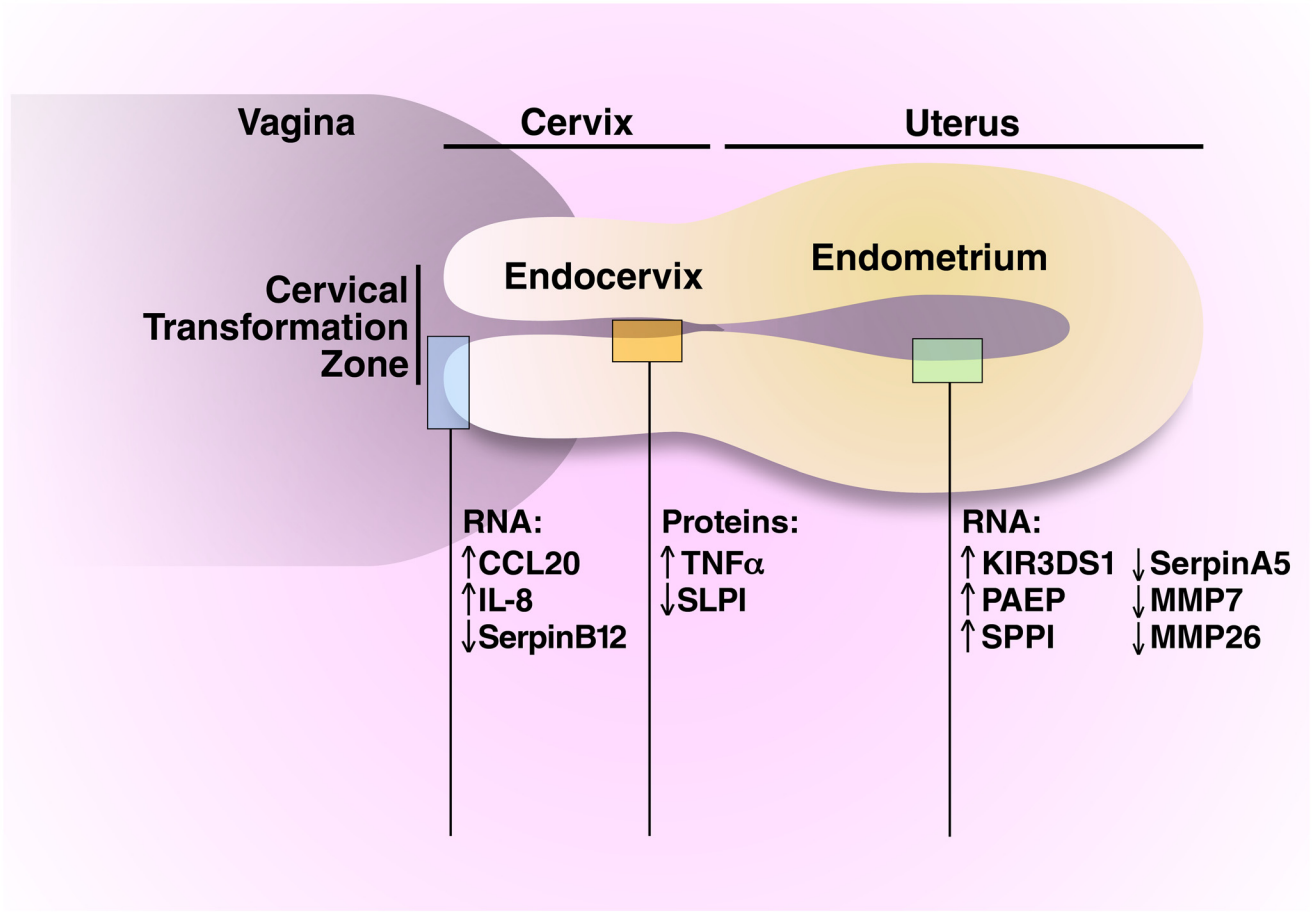
“Harm signal” of N9 and UPG on the upper FRT. This figure shows changes in gene and protein expression and functional pathways in the cervix, endocervix and endometrium that are perturbed by both N9 and UPG. All the changes represented in this figure were statistically significantly altered compared to the no-gel exposure arm with the exception of the protein concentrations of SLPI and TNFα in the UPG arm, in which the differences did not reach statistical significance.

The endocervix is the upper FRT site that is closest to the location of intravaginal gel application (Fig 1). To explore protein expression changes induced by N9 and UPG, we studied levels of cytokines, chemokines and innate immune factors in endocervical fluid extracted onto absorbant wicks (56 samples from 27 participants). The protein levels that we measured were similar to those in endocervical wick samples reported by others for IL6, IL8, IL10 and IL12, but our values were lower for IL1β and higher for IFNγ [26].

For N9 exposure compared with no-gel, SLPI protein concentration was significantly lower according to both statistical tests (p<0.05), while IL6 was significantly lower according to the parametric (chi-squared) but not the nonparametric (Wilcoxon) method (Table 4). The concentrations of 3 proteins, lactoferrin, MIP1α and IL1β, were significantly higher (p<0.05) according to both statistical tests used (Table 4). TNFα was significantly elevated following N9 exposure according to both statistical tests used when p≤0.10. IL1α levels were significantly higher (p<0.05) in the N9 arm compared to the no-gel arm according to parametric tests (chi-squared) but not nonparametric tests (Wilcoxon). Agreement between the nonparametric and parametric statistical tests was quite high. Discrepant signals between statistical methods arise because the single parametric model accounts for 1 to 3 exposures per person and generates tests of N9:no-gel and UPG:no-gel, while the Wilcoxon method is applied in two subsamples restricted to paired intervention:no-gel data. Discrepant signals also occur because the parametric model analyzes raw values while Wilcoxon analyzes the ranks of the values; and because the sample sizes are small.

**Table 4:** Concentrations of endocervical wick protein levels of cytokines, chemokines and innate immune factors, ordered by ratio of N9 versus no-gel levels. Horizontal gridlines divide the proteins into 3 categories: proteins whose concentrations are significantly down-regulated by N9 versus no-gel (ratio < 1; bold font); protein concentrations not significantly altered by N9 (ratio ≈ 1); and protein concentrations significantly up-regulated by N9 (ratio > 1, bold font).

	No-Gel Protein Concentration	N9 vs. No Gel			UPG vs. No Gel			N9 vs. UPG		
Cytokine/Chemokine/innate immune factor	Mean (95% CI), pg/ml	Mean Fold change (95% CI)	ChiSq^§^ p-value (# outcomes)	WSR^§^ p-value (# pairs)	Mean Fold change (95% CI)	ChiSq^§^ p-value (# outcomes)	WSR^§^ p-value (# pairs)	Mean Fold change (95% CI)	ChiSq^§^ p-value (# outcomes)	WSR^§^ p-value (# pairs)
IL6	**3.93 (2.29, 6.73) x10** ^**3**^	**0.47 (0.24, 0.92)**	**0.030 (38)**	**0.27 (14)**	**0.71 (0.36, 1.43)**	**0.33 (38)**	**0.90 (14)**	**0.66(0.33, 1.30)**	**0.22 (38)**	**0.30 (13)**
SLPI	**51.7 (33.9, 78.9)**	**0.53 (0.30, 0.92)**	**0.026 (25)**	**0.042 (12)**	**0.64 (0.36, 1.14)**	**0.12 (23)**	**0.21 (11)**	**0.82(0.47, 1.45)**	**0.49 (24)**	**0.76 (11)**
MCP1	5.61 (3.45, 9.12) x10^3^	0.84 (0.45, 1.56)	0.57 (38)	0.95 (14)	1.07 (0.57, 2.02)	0.82 (38)	0.63 (14)	0.78 (0.42, 1.46)	0.43 (38)	0.89 (13)
IL10	197 (102, 379)	1.09 (0.58, 2.03)	0.78 (38)	0.57 (14)	1.10 (0.59, 2.06)	0.76 (38)	0.73 (14)	1.06(0.53, 2.11)	0.86 (38)	0.76 (13)
IFNalpha2	233 (163, 332)	1.12 (0.71, 1.76)	0.61 (38)	0.10 (14)	1.13 (0.71, 1.81)	0.59 (38)	0.54 (14)	0.99(0.62, 1.57)	0.96 (38)	0.97 (12)
IL12	78.6 (43.5, 142)	1.21 (0.57, 2.57)	0.60 (38)	0.68 (14)	1.12 (0.52, 2.40)	0.77 (38)	0.54 (14)	1.00(0.40, 2.48)	1.00 (38)	0.91 (13)
IL8	70.2 (47.6, 104) x10^3^	1.27 (0.81, 2.01)	0.29 (38)	0.19 (14)	0.78 (0.49, 1.25)	0.30 (38)	0.36 (14)	1.62(1.02, 2.58)	0.040 (38)	0.24 (13)
MIP1beta	1.10 (0.64, 1.91) x10^3^	1.30 (0.68, 2.47)	0.42 (38)	0.10 (14)	1.30 (0.68, 2.47)	0.42 (38)	0.95 (14)	0.99(0.52, 1.90)	0.98 (38)	0.95 (13)
IFNgamma	47.9 (33.7, 68.0)	1.39 (0.91, 2.12)	0.12 (38)	0.024 (14)	1.30 (0.84, 2.01)	0.23 (38)	0.39 (14)	1.07(0.69, 1.65)	0.75	0.62 (12)
Human neutrophil peptides 1–3	7.16 (3.56, 14.4)	1.46 (0.76, 2.81)	0.24 (25)	0.30 (12)	1.22 (0.62, 2.41)	0.55 (23)	0.46 (11)	1.20 (061, 2.35)	0.58 (38)	0.52 (11)
RANTES	1.51 (0.73, 3.10) x10^3^	1.47 (0.62, 3.48)	0.37 (38)	0.50 (14)	0.87 (0.36, 2.08)	0.74 (38)	0.50 (14)	1.70(0.71, 4.05)	0.22 (38)	0.41 (13)
Lactoferrin	**585 (372, 920)**	**1.81 (1.07, 3.06)**	**0.030 (25)**	**0.052 (12)**	**1.22 (0.70, 2.11)**	**0.47 (23)**	**0.64 (11)**	**1.48 (0.86, 2.55)**	**0.14 (24)**	**0.41 (11)**
TNFalpha	**85.8 (47.4, 155)**	**1.95 (0.88, 4.34)**	**0.10 (38)**	**0.017 (14)**	**1.55 (0.68, 3.51)**	**0.29 (38)**	**0.22 (14)**	**1.26(0.57, 2.81)**	**0.56 (38)**	**0.74 (13)**
MIP1alpha	**784 (482, 1277)**	**2.00 (1.14, 3.53)**	**0.018 (38)**	**0.017 (14)**	**1.16 (0.65, 2.06)**	**0.60 (38)**	**0.67 (14)**	**1.73(0.97, 3.06)**	**0.061 (38)**	**0.34 (13)**
IL1alpha	**9.03 (5.02, 1.62) x10** ^**3**^	**2.91 (1.29, 6.53)**	**0.011 (38)**	**0.17 (14)**	**0.67 (0.29, 1.54)**	**0.34 (38)**	**0.95 (14)**	**4.32 (1.92, 9.71)**	**0.0006 (38)**	**0.24 (13)**
IL1beta	**1.06 (0.50, 2.25) x10** ^**3**^	**3.65 (1.34, 9.90)**	**0.012 (38)**	**0.017 (14)**	**1.07 (0.39, 2.99)**	**0.89 (38)**	**0.22 (14)**	**3.39 (1.24, 9.25)**	**0.018**	**0.34 (13)**

*56* Samples analyzed: N9, *n* = 20; UPG, *n* = 18; No gel, *n* = 18

^**§**^ ChiSquare (ChiSq) method includes subjects with outcomes following one or both exposures; Wilcoxon signed-rank (WSR) method includes subjects with outcomes following both exposures

For UPG exposures compared with no-gel, SLPI and IL6 concentrations were both decreased in the UPG arm, but the effects were not statistically significant. TNFα, which was significantly elevated in the N9 compared to the no-gel arm, was also higher in the UPG arm but did not reach statistical significance. Generally, outcomes did not differ between the N9 and UPG arms with the exception of IL8, IL1α and IL1β, which were significantly higher in the N9 arm (Table 4).

Finally, direct comparisons of N9 vs. UPG indicate that the effects of N9 on protein concentrations would be judged differently in 5 of 16 cases, if UPG were used as a control instead of no-gel. Of the 7 statistically significant N9:no-gel effects, the N9:UPG effect was nonsignificant in 4 cases, including SLPI and TNFα (UPG control would produce a false negative result). Of 9 N9:no-gel effects that were not statistically significant, the N9:UPG effect was significant in one case (UPG control would produce a false positive).

### Effects of N9 and UPG on phenotypic properties of T-cells from the endocervix

To examine the effects of the experimental conditions on the phenotypes of T-cells in the endocervix, fresh T-cells were isolated from endocervical curettage samples and analyzed by multiparameter flow cytometry (48 samples from 25 participants). T-cells expressing chemokine receptors CCR5 and CXCR4 and activation markers CD38 and HLA-DR were measured [27]. Memory T cell subsets were classified based on the expression of CCR7 and CD45RA as naïve (CCR7+CD45RA+), central memory (CCR7+CD45RA-), effector memory (CCR7-CD45RA-) and terminally differentiated effector memory (CCR7-CD45RA+) [27–29].

Relative to frequencies of CD4+ T-cell phenotypes in women in the no-gel arm, no statistically significant changes were observed following N9 or UPG exposure (S4 Table). However, the frequency of CD4+/X4+R5- cells was reduced in UPG-exposed compared to no-gel samples and approached statistical significance (fold-change: 0.59, 95% CI 0.33–1.07 p = 0.081).

For endocervical CD8+ T cells, the frequencies of CD8+ central memory (CCR7+CD45RA-) cells were significantly reduced by UPG exposure (fold-change: 0.52, 95% CI 0.29–0.96, p = 0.038; S5 Table). The frequencies of CD8+/CCR7-/CD45RA- (effector memory) cells and CD8+/X4+R5- cells were reduced in N9-exposed compared to no-gel samples and approached statistical significance (difference: -7.27 (-15.6, 1.01); p = 0.084 and fold-change: 0.66, 95% CI 0.41–1.06 p = 0.09, respectively).

### Effects of N9 and UPG on the transcriptome of the endometrium

The uterus is the organ furthest from the site of gel application (Fig 1). We used transcriptional profiling as a sensitive method to detect effects of intravaginal products on the endometrium. N9 exposure resulted in up (63 genes)-or down (30 genes)-regulation (≥1.5 fold, p<0.05) of 93 genes compared to no-gel samples (27 samples from 17 participants). The complete list of differentially expressed genes is presented in S6 Table. A partial list of genes with altered expression that were validated by PCR is presented in Table 2. N9 exposure was associated with up-regulation of several genes regulating immune function such as a member of the killer cell immunoglobulin-like receptor (KIR) gene family [30], glycodelin-A (PAEP) which induces IL6 secretion [31], and potent mediators of inflammation such as osteopontin (SPP1) [32] and phospholipase A2 group 2A (PLA2G2A) [33]. Pathway analysis did not reveal any significant effects of N9 on the endometrium.

UPG exposure resulted in up (211 genes)-or down (186 genes)-regulation (≥1.5 fold, p<0.05) of 397 genes in the endometrium compared to no-gel samples. This group had the largest number of gene expression changes found in our study. The complete list of differentially expressed genes is presented in S7 Table. A partial list of genes with altered expression that were validated by PCR is presented in Table 2. Several of the genes that demonstrated altered transcription were immune modulators such as complement component 3 (CC3) [34], glycodelin-A (PAEP) [31], osteopontin (SPP1) [32], CXCL13 [35], CCL21 [36], and a member of the KIR gene family [30]. Pathways associated with cell-to-cell signaling, cell death and survival, and cellular movement were altered and approached statistical significance (Z scores 1.5–2.0) in Ingenuity Pathway Analysis (Table 5). Many of the functions that were altered predicted increased adhesion and chemotaxis of potential HIV target cells including lymphocytes and monocytes.

**Table 5:** Top biofunctions affected by differential gene expression in endometrium biopsy samples from women using UPG compared to no gel. Biofunction analysis conducted through Ingenuity Pathway Analysis (IPA), Z scores ≥1.5. Pathways present in more than 1 category are represented only once.

Category	Disease or Functions	Predicted Activity	Z-score	Molecules	#*
Cardiovascular System Development and Function	development of blood vessel	Increased	1.59	ADAMTS8,AIMP1,ANGPTL1,C3,COL1A2,CXCR4,DKK1,EDNRB,FN1,HEY1,IGF2,IGHG1,MET,PROCR,PTGS2,RAMP2,SAA1,SLC8A1,TIMP3,TRPC4,XDH	21
Cell-To-Cell Signaling and Interaction	adhesion of lymphocytes	Increased	1.97	CCL21,FN1,IL6ST,SPP1,TLR4	5
	adhesion of mononuclear leukocytes	Increased	1.78	CCL21,FN1,IL15,IL6ST,SPP1,TLR4	6
	adhesion of blood cells	Increased	1.73	C3,CCL21,CXCR4,FGA,FN1,GZMB,IGHM,IL15,IL6ST,SAA1,SERPING1,SPP1,TLR4	13
	adhesion of immune cells	Increased	1.74	CCL21,CXCR4,FGA,FN1,IL15,IL6ST,SERPING1,SPP1,TLR4	9
Cell Death and Survival	apoptosis of synovial cells	Increased	1.96	CLU,FN1,SAA1,TIMP3	4
	cell viability of leukocytes	Increased	1.71	CLU,CXCR4,FN1,IL15,IL2RB,TLR4	6
	cell death of connective tissue cells	Increased	1.63	CLU,COMP,ESR1,FN1,GSN,MAP3K5,SAA1,TIMP3,XDH	9
Cellular Movement	chemotaxis of leukocytes	Increased	1.73	C3,CCL21,CXCL13,CXCR4,EDN3,EDNRB,IL15,SAA1,SPP1,TLR4	10
Organismal Development	vasculogenesis	Increased	1.59	ADAMTS8,AIMP1,ANGPTL1,C3,CXCR4,DKK1,EDNRB,FN1,HEY1,IGF2,IGHG1,MET,PROCR,PTGS2,RAMP2,SAA1,SLC8A1,TIMP3,TRPC4,XDH	20

* number of molecules

### Effects of N9 and UPG on phenotypic properties of T-cells from the endometrium

To examine the effects of the experimental conditions on the phenotypes of T-cells in the uterus, fresh T-cells were isolated from endometrial biopsies and analyzed by multiparameter flow cytometry (36 samples from 19 participants). Relative to frequencies of CD4+ and CD8+ T-cell phenotypes in women in the no-gel arm, no statistically significant elevations or suppressions of the frequencies were observed in the endometrium following N9 and UPG exposure (S4 and S5 Tables). The frequency of CD4+ central memory (CCR7+CD45RA-) cells was increased in the arm exposed to UPG compared to no-gel (S4 Table) and approached statistical significance (1.59 95% CI 0.96–2.64, p<0.10). The frequency of CD8+ central memory (CCR7+CD45RA-) cells was decreased in the arm exposed to UPG compared to no-gel (S5 Table), similar to what was observed in the endocervix, and approached statistical significance (0.54 95% CI 0.26–1.11, p<0.10).

Comparing the frequencies of cell phenotypes in endocervical curettage tissue to endometrial biopsies, many significant differences were observed (S4 and S5 Tables). For both CD4+ and CD8+ T cells, effector memory and R5+ phenotypes were significantly enriched in endometrium compared to endocervix, consistent with previously reported results from a subset of this sample [37].

### A common “harm signal” of N9 and UPG effects (Fig 1)

Effects on the cervix and upper FRT that are common to both N9 and UPG might be helpful in defining a “harm signature” that can be used in safety studies of new microbicides. At the cervical TZ, both N9 and UPG resulted in a common transcriptome “harm signal” with up-regulation of CCL20 and IL8 (Fig 1). These genes are up-regulated as an early event after infection in a simian model of HIV infection, resulting in recruitment of target T-cells and amplification of infection [38]. Both N9 and UPG resulted in down-regulated RNA expression of SERPINB12; other family members of the SERPIN family are important in protection against HIV infection [39].

In the endocervix, both N9 and UPG resulted in decreased protein levels in endocervix of SLPI, which protects against HIV infection [40], and with increased levels of inflammatory agents TNFα, although the differences were not statistically significant for UPG.

In the endometrium, both N9 and UPG resulted in a common transcriptomic “harm signal” with up-regulation of KIR3DS1, glycodelin-A (PAEP) and osteopontin (SPP1), and down-regulation of SERPINA5, MMP7 and MMP26. Glycodelin-A and osteopontin can function as cytokine and immune cell modulators, as discussed above. The decreased expression of SERPINA5 suggests possible compromised protection against HIV infection afforded by other SERPIN family members through inhibition of HIV proteases [39]. Ingenuity Pathway Analysis indicated that N9 (in the cervical TZ) and UPG (in the uterus) altered pathways that function to increase adhesion and chemotaxis of potential HIV target cells.

## Discussion

This study was designed to explore the *in vivo* effects of the intravaginal products N9 and UPG on the upper FRT. We observed that both N9 and UPG produced significant inflammatory effects after intravaginal administration *in vivo*, as measured by several experimental parameters. The presence of inflammation in either the FRT or in semen is thought to increase the risk of heterosexual HIV transmission [41]. Thus, unmeasured inflammatory effects of intravaginal products on the upper FRT may have blunted the potentially beneficial effects of various products in clinical trials of HIV prevention.

One goal of our study was designed to determine whether the harmful effects of N9 noted in the lower FRT were also observed in the upper FRT. We found that N9 impacted all of the experimental parameters measured. Exposure to N9 resulted in resulted in up-regulation of both RNA (cervical TZ) and protein (endocervix) for IL1α and IL1β. Similar increases of IL1α and IL1β by N9 have been reported in cervicovaginal lavage (CVL) samples from women after N9 use [42]. The same group also reported increased levels of IL8 in CVL fluids, consistent with our finding that IL8 RNA was increased in the cervical TZ after N9 exposure, although we did not detect significantly increased IL8 protein levels in endocervical fluid. We found that N9 exposure resulted in lower protein levels in endocervical fluid of SLPI, a protease inhibitor that is part of the innate immune response for protection of epithelium against damage and that directly blocks HIV infection of target T-cells [40]. Other groups have observed a reduction in SLPI in the CVLs of women exposed to N9 [7,42]. The concordance of findings in endocervical and CVL fluids supports our hypothesis that intravaginally-applied N9 gains access to the upper FRT and has similar effects there as have been observed in the lower FRT.

Exposure to N9 also altered gene expression of inflammatory mediators in the endometrium. Another study found that intravaginal N9 resulted in reduced endometrial levels of IL8 and IL1β [43], supporting our findings that N9 perturbs the endometrium although we did not observe altered expression of those specific genes in our study. In cases where the effects could be directly compared between sites (pathway analysis and T-cell phenotypes), the effects of N9 were stronger on the sites closer to the vagina (cervical TZ or endocervix) than those further away (endometrium), consistent with a dose effect. Our results support a model in which the known pathological effects of N9 on the lower FRT extend into both the endocervix and the endometrium and may facilitate HIV infection in the upper FRT.

Our results demonstrated that UPG also altered the immune microenvironment of the upper FRT. At the cervical TZ, UPG resulted in increased RNA expression of inflammatory mediators IL8 and CCL20. In the endocervix, UPG resulted in decreased protein levels of SLPI, potentially weakening the innate immune protection against HIV, although the result was not statistically significant. Another study found that protein levels of IL8 did not change and SLPI increased initially in CVLs from women using UPG [42]; the differences between the results from their study and ours may reflect different sites of sample collection, product use frequency (twice versus once daily) and for IL8, measurement of protein versus RNA. In the endometrium, exposure to UPG resulted in up-regulation of large numbers of genes contributing to processes of immune cell trafficking and cell death/survival. Complement component 3, which had increased RNA levels after exposure to UPG, has been implicated in enhancement of HIV-1 infectivity of DCs and epithelial cells [44,45].

Our surprising findings of inflammatory effects of UPG on the endocervix and endometrium underscore the need to consider the effects of gel excipients on the upper FRT. UPG was tested for safety and acceptability in women using measurements from the lower FRT [11]. UPG did not contribute to an increased risk of HIV infection compared to women not using gel in the HPTN-035 microbicide trial [46]. However, if a microbicide vehicle has inflammatory effects, it may counteract the protective effect of antiretroviral agents present in microbicide gels. Differences in vehicles used to deliver tenofovir may account for the protective effect of 1% tenofovir gel seen in the CAPRISA trial [9] but absent in the VOICE trial [10], although low adherence to product use also likely contributed to the trial failure. UPG was used as the placebo gel in both trials due to its isotonic properties, lack of anti-viral activity, and proven acceptability [11,47]. However, some gels have been shown to exhibit toxicity on epithelial surfaces [48]; if UPG has a similar effect, this may explain some of the findings reported herein.

A unique feature of this study is the use of multiple experimental platforms to test gel effects including measurements of RNA, protein and T-cell phenotypes. This is first study to apply gene expression profiling of the upper FRT to study microbicide effects, and the results indicate that the transcriptome provides a panel of gene expression changes that will be useful for identifying a “harm signal” for other intravaginal interventions. Our results indicate that protein measurements from endocervical wicks provide a non-invasive method for identifying pro-inflammatory effects. Another unique strength of our approach is the timing of sample collection to fall within a narrow window of the menstrual cycle thus limiting variability from the hormonal fluctuations within the menstrual cycle, and timed to coincide with the “window of vulnerability” for HIV infection [13]. Finally, since the randomized crossover study design exposes participants to multiple interventions, participants are exposed to study arms in a random order and intervention effects relative to control are estimated within participants. This design reduces bias by controlling for personal characteristics that might affect outcomes, ensures balanced sample sizes across arms, and increases power to detect significant effects relative to a parallel-arm design.

The main limitation of this study is the relatively small sample size and the unwillingness of some participants to complete all 3 study cycles; it highlights the challenge of recruiting healthy volunteers to undergo uncomfortable procedures for the sake of research. Many of the outcome measurements were based on small sample sizes and the results need to be further validated in larger studies. While participants were instructed to refrain from sexual intercourse for 72 hours prior to biopsy collection, and were asked to confirm that they had done so at the time of the biopsy visit, it is possible that unreported sexual intercourse might have contributed to some of the results observed since we did not test for the presence of semen. Another limitation is that *in vivo* effects of N9 and UPG on HIV infectivity of cells in the FRT were not studied directly. However, studies with endometrial, endocervical and HIV target cells *in vitro* can provide further insights into the processes by which these agents perturb the upper FRT and might enhance HIV risk.

To translate these findings to the clinic, the upper FRT should be included in safety evaluations of vaginal products, particularly those that are likely to be used by women at increased risk for exposure to HIV. The finding of a common N9 and UPG “harm signal” on the upper FRT provides a paradigm for safety testing of future HIV prevention strategies. Our results challenge the existing paradigm for safety assessments of candidate microbicides that focus only on the lower FRT, and indicate that the development of a truly inactive vaginal placebo gel is needed. Clinical trials of microbicide candidates should consider the inclusion of a “no-gel” control arm until biologically inert placebo products are developed. Our findings therefore have broad implications for safety testing of future candidate microbicides and for consideration of the upper reproductive tract as a possible portal for HIV acquisition.

## Supporting Information

S1 Table Numbers of samples per specimen type and study exposure group that contributed to each analysis. (DOCX)

S2 Table The complete list of differentially expressed genes in N9-exposed cervix compared to unexposed cervix (p<0.05, fold change ≥1.5). (DOCX)

S3 Table The complete list of differentially expressed genes in UPG-exposed cervix compared to unexposed cervix (p<0.05, fold change ≥1.5). (DOCX)

S4 Table CD4+ T-cell phenotypes in samples from the endocervix and endometrium: (i) percentages in the group with no-gel exposure, (ii) relative effects after exposure to intravaginal N9 or UPG and (iii) relative frequencies, endocervix versus endometrium, in the group with no-gel exposure. For analyses conducted on the log scale, relative effects represent ratios of groups (normal font), whereas for analyses conducted on the decimal scale, relative effects represent differences between groups ***(bold*, *italic font)***.
(DOCX)

S5 Table CD8+ T-cell phenotypes in samples from the endocervix and endometrium: (i) percentages in the group with no-gel exposure, (ii) relative effects after exposure to intravaginal N9 or UPG and (iii) relative frequencies, endocervix versus endometrium, in the group with no-gel exposure. For analyses conducted on the log scale, relative effects represent ratios of groups (normal font), whereas for analyses conducted on the decimal scale, relative effects represent differences between groups ***(bold*, *italic font)***.
(DOCX)

S6 Table The complete list of differentially expressed genes in N9-exposed endometrium compared to unexposed endometrium (p<0.05, fold change ≥1.5). (DOCX)

S7 Table The complete list of differentially expressed genes in UPG-exposed endometrium compared to unexposed endometrium (p<0.05, fold change ≥1.5). (DOCX)
