# Supplementary material for: Unexpected Inflammatory Effects of Intravaginal Gels (Universal Placebo Gel and Nonoxynol-9) on the Upper Female Reproductive Tract: A Randomized Crossover Study
Source: PLoS One. 2015 Jul 15;10(7):e0129769. doi: 10.1371/journal.pone.0129769 (PMC4503751; doi:10.1371/journal.pone.0129769)
Supplement: S1 Table — (DOCX) [file pone.0129769.s001.docx]

**S1 Table**. **Numbers of samples per specimen type and study exposure group that contributed to each analysis**

**Endocervical wick for protein analysis (Table 4)^1^**

| # Exposures  Per woman | Exposure Group | | | Cumulative | Pairs available | |
| --- | --- | --- | --- | --- | --- | --- |
|  | N9 | UPG | No Gel | Exposures / Women | N9:Unt | UPG:Unt |
| 1 | 5 | 3 | 2 | 10 / 10 | 0 | 0 |
| 2 | 3 | 3 | 4 | 10 /  5 | 2 | 2 |
| 3 | 12 | 12 | 12 | 36 / 12 | 12 | 12 |
| Any | 20 | 18 | 18 | 56 / 27 | 14 | 14 |

**Cervical curettage specimens for T cell phenotying  (S4 and S5 Tables) ^2^**

| # Exposures  Per woman | Exposure Group | | | Cumulative | Pairs available | |
| --- | --- | --- | --- | --- | --- | --- |
|  | N9 | UPG | No Gel | Exposures / Women | N9:Unt | UPG:Unt |
| 1 | 5 | 3 | 2 | 10 / 10 | 0 | 0 |
| 2 | 4 | 4 | 6 | 14 / 7 | 3 | 3 |
| 3 | 8 | 8 | 8 | 24 / 8 | 8 | 8 |
| Any | 17 | 15 | 16 | 48 / 25 | 11 | 11 |

**Endometrial biopsy specimens for T cell phenotying  (S4 and S5 Tables) ^2^**

| # Exposures  Per woman | Exposure Group | | | Cumulative | Pairs available | |
| --- | --- | --- | --- | --- | --- | --- |
|  | N9 | UPG | No Gel | Exposures / Women | N9:Unt | UPG:Unt |
| 1 | 3 | 3 | 2 | 8 / 8 | 0 | 0 |
| 2 | 4 | 3 | 5 | 12 / 6 | 3 | 2 |
| 3 | 5 | 5 | 5 | 15 / 5 | 5 | 5 |
| Any | 12 | 11 | 12 | 35 / 19 | 8 | 7 |

**Cervical biopsy specimens for transcriptional profiling (Tables 2 and 3) ^3^**

| # Exposures  Per woman | Exposure Group | | | Cumulative | Pairs available | |
| --- | --- | --- | --- | --- | --- | --- |
|  | N9 | UPG | No Gel | Exposures / Women | N9:Unt | UPG:Unt |
| 1 | 5 | 2 | 4 | 11 / 11 | 0 | 0 |
| 2 | 2 | 3 | 5 | 10 / 5 | 2 | 3 |
| 3 | 4 | 4 | 5 | 15 / 5 | 5 | 5 |
| Any | 12 | 10 | 12 | 36 / 21 | 7 | 8 |

**Endometrial biopsy specimens for transcriptional profiling (Tables 2 and 3) ^3^**

| # Exposures  Per woman | Exposure Group | | | Cumulative | Pairs available | |
| --- | --- | --- | --- | --- | --- | --- |
|  | N9 | UPG | No Gel | Exposures / Women | N9:Unt | UPG:Unt |
| 1 | 3 | 3 | 4 | 10 / 10 | 0 | 0 |
| 2 | 3 | 3 | 2 | 8 / 4 | 1 | 1 |
| 3 | 3 | 3 | 3 | 9 / 3 | 3 | 3 |
| Any | 9 | 9 | 9 | 27 / 17 | 4 | 4 |

^1^ samples were excluded if the volume of fluid recovered from the wick was <2 ml (n=1)

^2^ samples were excluded if the number of CD4+ or CD8+ T-cells was less than 500 (n=1 for curettage, n=0 for endometrial biopsy)

^3^ samples were excluded due to low RIN number/RNA degradation (n=7 for cervix and n=6 for endometrium)
