# Supplementary material for: Unexpected Inflammatory Effects of Intravaginal Gels (Universal Placebo Gel and Nonoxynol-9) on the Upper Female Reproductive Tract: A Randomized Crossover Study
Source: PLoS One. 2015 Jul 15;10(7):e0129769. doi: 10.1371/journal.pone.0129769 (PMC4503751; doi:10.1371/journal.pone.0129769)
Supplement: S2 Table — (DOCX) [file pone.0129769.s002.docx]

**S2 Table. The complete list of differentially expressed genes in N9-exposed cervix compared to unexposed cervix (p<0.05, fold change ≥1.5)**

| **Gene Description** | **Gene Symbol** | **Fold Change** | **Regulation** |
| --- | --- | --- | --- |
| chitinase 3-like 1 (cartilage glycoprotein-39) | CHI3L1 | 2.21 | up |
| interleukin 8 | IL8 | 2.18 | up |
| amphiregulin | AREG | 2.18 | up |
| chemokine (C-C motif) ligand 20 | CCL20 | 2.11 | up |
| amphiregulin | AREG | 2.05 | up |
| keratin 17 | KRT17 | 2.01 | up |
| interleukin 1, alpha | IL1A | 1.91 | up |
| selectin E | SELE | 1.87 | up |
| heparin-binding EGF-like growth factor | HBEGF | 1.87 | up |
| interleukin 1, beta | IL1B | 1.82 | up |
| keratin 17 | KRT17 | 1.81 | up |
| chemokine (C-C motif) ligand 19 | CCL19 | 1.80 | up |
| interleukin 1 family, member 9 | IL1F9 | 1.79 | up |
| cholesterol 25-hydroxylase | CH25H | 1.68 | up |
| chemokine (C-X-C motif) ligand 2 | CXCL2 | 1.64 | up |
| apolipoprotein B mRNA editing enzyme, catalytic polypeptide-like 3A | APOBEC3A | 1.64 | up |
| keratin 17 | KRT17 | 1.61 | up |
| fatty acid binding protein 4, adipocyte | FABP4 | 1.58 | up |
| interleukin 6 (interferon, beta 2) | IL6 | 1.57 | up |
| tumor necrosis factor receptor superfamily, member 12A | TNFRSF12A | 1.55 | up |
| immediate early response 3 | IER3 | 1.54 | up |
| carbonic anhydrase II | CA2 | 1.53 | up |
| fibroblast activation protein, alpha | FAP | 1.53 | up |
| myosin IB | MYO1B | 1.52 | up |
| ornithine decarboxylase 1 | ODC1 | 1.52 | up |
| glycerol kinase \| glycerol kinase 3 pseudogene | GK\|GK3P | 1.51 | up |
| chemokine (C-C motif) ligand 2 | CCL2 | 1.51 | up |
| solute carrier family 7, (cationic amino acid transporter, y+ system) member 11 | SLC7A11 | 1.51 | up |
| glycerol kinase | GK | 1.51 | up |
| immediate early response 3 | IER3 | 1.50 | up |
| immediate early response 3 | IER3 | 1.50 | up |
| cytochrome P450, family 24, subfamily A, polypeptide 1 | CYP24A1 | 1.50 | up |
| epiregulin | EREG | 1.50 | up |
| retinoic acid early transcript 1L | RAET1L | 1.50 | down |
| clusterin | CLU | 1.51 | down |
| amylase, alpha 2A (pancreatic) \| amylase, alpha 1A (salivary) \| amylase, alpha 1B (salivary) \| amylase, alpha 1C (salivary) \| amylase, alpha 2B (pancreatic) | AMY2A\|AMY1A\|AMY1B\|AMY1C\|AMY2B | 1.51 | down |
| keratin 10 | KRT10 | 1.51 | down |
| premature ovarian failure, 1B | POF1B | 1.51 | down |
| periplakin | PPL | 1.51 | down |
| amylase, alpha 1A (salivary) \| amylase, alpha 1B (salivary) \| amylase, alpha 1C (salivary) \| amylase, alpha 2A (pancreatic) \| amylase, alpha 2B (pancreatic) | AMY1A\|AMY1B\|AMY1C\|AMY2A\|AMY2B | 1.51 | down |
| amylase, alpha 1A (salivary) \| amylase, alpha 1B (salivary) \| amylase, alpha 1C (salivary) \| amylase, alpha 2A (pancreatic) \| amylase, alpha 2B (pancreatic) | AMY1A\|AMY1B\|AMY1C\|AMY2A\|AMY2B | 1.52 | down |
| amylase, alpha 1A (salivary) \| amylase, alpha 1B (salivary) \| amylase, alpha 1C (salivary) \| amylase, alpha 2A (pancreatic) \| amylase, alpha 2B (pancreatic) | AMY1A\|AMY1B\|AMY1C\|AMY2A\|AMY2B | 1.52 | down |
|  |  | 1.52 | down |
| small proline-rich protein 2E | SPRR2E | 1.52 | down |
| transmembrane protease, serine 11D | TMPRSS11D | 1.52 | down |
| non-specific cytotoxic cell receptor protein 1 homolog (zebrafish) | NCCRP1 | 1.53 | down |
| death associated protein-like 1 | DAPL1 | 1.53 | down |
| carcinoembryonic antigen-related cell adhesion molecule 5 | CEACAM5 | 1.53 | down |
| chromosome 10 open reading frame 99 | C10orf99 | 1.54 | down |
| epithelial membrane protein 1 | EMP1 | 1.54 | down |
| allograft inflammatory factor 1-like | AIF1L | 1.55 | down |
| solute carrier family 15 (oligopeptide transporter), member 1 | SLC15A1 | 1.55 | down |
| late cornified envelope 3A | LCE3A | 1.56 | down |
| family with sequence similarity 3, member B | FAM3B | 1.56 | down |
| small proline-rich protein 2B | SPRR2B | 1.56 | down |
| serpin peptidase inhibitor, clade B (ovalbumin), member 13 | SERPINB13 | 1.56 | down |
| small proline-rich protein 2A \| small proline-rich protein 2B | SPRR2A\|SPRR2B | 1.56 | down |
| small proline-rich protein 2G \| small proline-rich protein 2C (pseudogene) | SPRR2G\|SPRR2C | 1.57 | down |
| kallikrein-related peptidase 6 | KLK6 | 1.58 | down |
| sciellin | SCEL | 1.60 | down |
| chromosome 15 open reading frame 48 \| microRNA 147b | C15orf48\|MIR147B | 1.60 | down |
| interleukin 18 (interferon-gamma-inducing factor) | IL18 | 1.60 | down |
| solute carrier family 34 (sodium phosphate), member 2 | SLC34A2 | 1.61 | down |
| flavin containing monooxygenase 2 (non-functional) | FMO2 | 1.61 | down |
| uroplakin 1A | UPK1A | 1.61 | down |
| keratinocyte differentiation-associated protein | KRTDAP | 1.61 | down |
| Rh family, C glycoprotein | RHCG | 1.61 | down |
| desmoglein 1 | DSG1 | 1.61 | down |
|  | CD177 | 1.62 | down |
| calmodulin-like 5 | CALML5 | 1.62 | down |
| secreted LY6/PLAUR domain containing 1 | SLURP1 | 1.62 | down |
| small proline-rich protein 2D \| small proline-rich protein 2B | SPRR2D\|SPRR2B | 1.64 | down |
| keratin 4 | KRT4 | 1.65 | down |
| protein phosphatase 1, regulatory (inhibitor) subunit 3C | PPP1R3C | 1.65 | down |
| cytochrome P450, family 2, subfamily B, polypeptide 7 pseudogene 1 | CYP2B7P1 | 1.66 | down |
| protein disulfide isomerase family A, member 6 \| ATPase, H+ transporting, lysosomal 42kDa, V1 subunit C2 | PDIA6\|ATP6V1C2 | 1.67 | down |
| carcinoembryonic antigen-related cell adhesion molecule 6 (non-specific cross reacting antigen) | CEACAM6 | 1.69 | down |
|  | LOC441178 | 1.69 | down |
|  | LOC441178 | 1.70 | down |
| hydroxyprostaglandin dehydrogenase 15-(NAD) | HPGD | 1.70 | down |
| secretoglobin, family 3A, member 1 | SCGB3A1 | 1.71 | down |
| cysteine-rich C-terminal 1 | CRCT1 | 1.71 | down |
| cystatin E/M | CST6 | 1.71 | down |
| cornifelin | CNFN | 1.72 | down |
| cornulin | CRNN | 1.73 | down |
| tumor protein p63 regulated 1 | TPRG1 | 1.73 | down |
| mucin 21, cell surface associated | MUC21 | 1.74 | down |
| prostate stem cell antigen | PSCA | 1.74 | down |
|  | CD177 | 1.75 | down |
| prostate stem cell antigen | PSCA | 1.76 | down |
| ERO1-like (S. cerevisiae) | ERO1L | 1.77 | down |
| surfactant associated 2 | SFTA2 | 1.77 | down |
| surfactant associated 2 | SFTA2 | 1.78 | down |
| alpha-2-macroglobulin-like 1 | A2ML1 | 1.78 | down |
| serine peptidase inhibitor, Kazal type 5 | SPINK5 | 1.82 | down |
| mucin 21, cell surface associated | MUC21 | 1.88 | down |
| mucin 21, cell surface associated | MUC21 | 1.93 | down |
| interleukin 1 family, member 6 (epsilon) | IL1F6 | 1.94 | down |
| keratin 78 | KRT78 | 1.98 | down |
| uroplakin 1A | UPK1A | 2.00 | down |
| transmembrane protease, serine 11E | TMPRSS11E | 2.00 | down |
| transmembrane protease, serine 11E | TMPRSS11E | 2.01 | down |
| family with sequence similarity 25, member B \| family with sequence similarity 25, member C \| family with sequence similarity 25, member G \| family with sequence similarity 25, member A | FAM25B\|FAM25C\|FAM25G\|FAM25A | 2.03 | down |
| family with sequence similarity 25, member B \| family with sequence similarity 25, member C \| family with sequence similarity 25, member G \| family with sequence similarity 25, member A | FAM25B\|FAM25C\|FAM25G\|FAM25A | 2.03 | down |
| family with sequence similarity 25, member B \| family with sequence similarity 25, member C \| family with sequence similarity 25, member G \| family with sequence similarity 25, member A | FAM25B\|FAM25C\|FAM25G\|FAM25A | 2.03 | down |
| carcinoembryonic antigen-related cell adhesion molecule 7 | CEACAM7 | 2.26 | down |
| lymphocyte antigen 6 complex, locus G6C | LY6G6C | 2.32 | down |
| ribonuclease, RNase A family, 7 | RNASE7 | 2.36 | down |
| repetin | RPTN | 2.36 | down |
| lymphocyte antigen 6 complex, locus G6C | LY6G6C | 2.41 | down |
| lymphocyte antigen 6 complex, locus G6C | LY6G6C | 2.43 | down |
| transmembrane protease, serine 11B | TMPRSS11B | 2.46 | down |
| serpin peptidase inhibitor, clade B (ovalbumin), member 12 | SERPINB12 | 2.49 | down |
| mal, T-cell differentiation protein | MAL | 2.61 | down |
| serine peptidase inhibitor, Kazal type 7 (putative) | SPINK7 | 2.65 | down |
| keratinocyte proline-rich protein | KPRP | 2.88 | down |
| keratin 1 | KRT1 | 3.06 | down |
| late cornified envelope 3E | LCE3E | 3.86 | down |
| late cornified envelope 3D | LCE3D | 6.04 | down |
