# Supplementary material for: Unexpected Inflammatory Effects of Intravaginal Gels (Universal Placebo Gel and Nonoxynol-9) on the Upper Female Reproductive Tract: A Randomized Crossover Study
Source: PLoS One. 2015 Jul 15;10(7):e0129769. doi: 10.1371/journal.pone.0129769 (PMC4503751; doi:10.1371/journal.pone.0129769)
Supplement: S3 Table — (DOCX) [file pone.0129769.s003.docx]

**S3 Table. The complete list of differentially expressed genes in UPG-exposed cervix compared to unexposed cervix (p<0.05, fold change ≥1.5)**

| **Gene Description** | **Gene Symbol** | **Fold Change** | **Regulation** |
| --- | --- | --- | --- |
| chemokine (C-C motif) ligand 20 | CCL20 | 1.89 | up |
| cytochrome P450, family 2, subfamily B, polypeptide 7 pseudogene 1 | CYP2B7P1 | 1.61 | up |
| solute carrier family 26, member 4 | SLC26A4 | 1.60 | up |
| chromosome 20 open reading frame 114 | C20orf114 | 1.57 | up |
| olfactomedin 4 | OLFM4 | 1.56 | up |
| palate, lung and nasal epithelium associated | PLUNC | 1.53 | up |
| interleukin 8 | IL8 | 1.53 | up |
| chemokine (C-X-C motif) ligand 5 | CXCL5 | 1.50 | up |
| lymphocyte antigen 6 complex, locus G6C | LY6G6C | 1.63 | down |
| serpin peptidase inhibitor, clade B (ovalbumin), member 12 | SERPINB12 | 1.66 | down |
| lymphocyte antigen 6 complex, locus G6C | LY6G6C | 1.67 | down |
| lymphocyte antigen 6 complex, locus G6C | LY6G6C | 1.68 | down |
| keratin 10 | KRT10 | 1.96 | down |
| keratin 1 | KRT1 | 2.05 | down |
