# Supplementary material for: Unexpected Inflammatory Effects of Intravaginal Gels (Universal Placebo Gel and Nonoxynol-9) on the Upper Female Reproductive Tract: A Randomized Crossover Study
Source: PLoS One. 2015 Jul 15;10(7):e0129769. doi: 10.1371/journal.pone.0129769 (PMC4503751; doi:10.1371/journal.pone.0129769)
Supplement: S4 Table — For analyses conducted on the log scale, relative effects represent ratios of groups (normal font), whereas for analyses conducted on the decimal scale, relative effects represent differences between groups (bold, italic font). (DOCX) [file pone.0129769.s004.docx]

**S4 Table.** CD4+ T-cell phenotypes in samples from the endocervix and endometrium: (i) percentages in the group with no-gel exposure, (ii) relative effects after exposure to intravaginal N9 or UPG and (iii) relative frequencies, endocervix versus endometrium, in the group with no-gel exposure. For analyses conducted on the log scale, relative effects represent ratios of groups (normal font), whereas for analyses conducted on the decimal scale, relative effects represent differences between groups ***(bold, italic font).***

| CD4+ T-Cell phenotype |  | Endocervix | | | Endometrium | | | Cervix:Endom |
| --- | --- | --- | --- | --- | --- | --- | --- | --- |
|  | Analysis scale | ***(i) No Gel*** Mean (95% CI) | ***(ii) N9:No Gel*** Mean (95% CI) | ***(ii) UPG:No Gel*** Mean (95% CI) | ***(i) No Gel*** Mean (95% CI) | ***(ii) N9:No Gel*** Mean (95% CI) | ***(ii) UPG:No Gel*** Mean (95% CI) | ***(iii)*** ***No gel*** Mean (95% CI) |
| CD4+/CCR7+ | Log | 46.3  (34.9, 61.4) | 1.01  (0.68, 1.50) | 1.01  (0.76, 1.51) | 11.5  (8.30, 15.9) | 1.31  (0.82, 2.07) | 1.55  (0.97, 2.48) ‡ | 4.02  (2.61, 6.19) † |
| CD4+/CD45RA+ | Log | 11.1  (7.59, 16.1) | 1.28  (0.86, 1.90) | 1.00  (0.67, 1.50) | 3.12  (2.06, 4.72) | 0.81  (0.51, 1.29) | 0.93  (0.57, 1.49) | 3.54  (2.33, 5.40) † |
| CD4+/CCR7-/CD45RA-  (Effector Memory) | Log | 54.7  (49.3, 60.6) | 0.95  (0.83, 1.08) | 0.96  (0.84, 1.10) | 90.1  (80.1, 101) | 0.90  (0.78, 1.05) | 0.93  (0.80, 1.09) | 0.61  (0.53, 0.70) † |
| CD4+/CCR7-/CD45RA+  (Terminally Differentiated Effector) | Log | 3.18  (2.22, 4.54) | 1.20  (0.81, 1.78) | 1.11  (0.74, 1.67) | 2.42  (1.62, 3.60) | 0.72  (0.45, 1.14) | 0.91  (0.56, 1.46) | 1.31  (0.86, 2.01) |
| CD4+/CCR7+/CD45RA-  (Central Memory) | Log | 36.4  (26.8, 49.6) | 0.98  (0.64, 1.50) | 0.98  (0.63, 1.51) | 10.6  (7.44, 15.2) | 1.37  (0.84, 2.25) | 1.59  (0.96, 2.64) ‡ | 3.43  (2.16, 5.44) † |
| CD4+/CCR7+/CD45RA+  (Naïve) | Log | 8.44  (5.88, 12.1) | 1.15  (0.76, 1.76) | 0.98  (0.64, 1.51) | 1.84  (1.23, 2.77) | 1.15  (0.70, 1.87) | 1.03  (0.62, 1.70) | 4.58  (2.92, 7.17) † |
| CD4+/CD38+ | Log | 45.8  (40.5, 51.9) | 1.04  (0.89, 1.21) | 1.02  (0.87, 1.20) | 83.6  (72.5, 96.4) | 0.93  (0.77, 1.12) | 0.94  (0.78, 1.14) | 0.55  (0.46, 0.65) † |
| CD4+/HLADR+ | Log | 5.96  (3.97, 8.97) | 1.30  (0.80, 2.11) | 0.86  (0.52, 1.41) | 29.9  (18.9, 47.4) | 0.81  (0.46, 1.43) | 0.82  (0.46, 1.48) | 0.20  (0.12, 0.34) † |
| CD4+/CD38+HLADR+ | Log | 3.20  (2.11, 4.83) | 1.30  (0.77, 2.18) | 0.98  (0.58, 1.67) | 27.0  (16.8, 43.2) | 0.61  (0.33, 1.13) | 0.75  (0.40, 1.39) | 0.12  (0.07, 0.21) † |
| CD4+/CD38+HLADR- | Log | 42.4  (37.1, 48.5) | 1.00  (0.84, 1.18) | 1.01  (0.85, 1.20) | 59.3  (50.9, 69.2) | 1.00  (0.83, 1.22) | 0.97  (0.79, 1.19) | 0.72  (0.60, 0.86) † |
| CD4+/CD38-HLADR+ | Log | 3.62  (2.76, 4.75) | 1.03  (0.74, 1.42) | 0.85  (0.61, 1.18) | 2.82  (2.08, 3.83) | 1.03  (0.71, 1.50) | 1.00  (0.68, 1.48) | 1.29  (0.91, 1.81) |
| CD4+/X4+ | Decimal | ***44.4***  ***(34.0, 54.8)*** | ***-5.94***  ***(-18.4, 6.55)*** | ***-5.98***  ***(-18.7, 6.76)*** | ***24.6***  ***(12.9, 36.4)*** | ***2.70***  ***(-11.9, 17.3)*** | ***-2.11***  ***(-17.1, 12.9)*** | ***19.8***  ***(6.40, 33.2) ¶*** |
| CD4+/R5+ | Decimal | ***26.4***  ***(16.2, 36.7)*** | ***0.97***  ***(-11.8, 13.8)*** | ***-5.04***  ***(-18.1, 8.05)*** | ***66.2***  ***(54.5, 77.9)*** | ***4.57***  ***(-10.4, 19.5)*** | ***-3.55***  ***(-11.8, 19.0)*** | ***-39.8***  ***(-53.5, -26.0)*** † |
| CD4+/X4+R5+ | Log | 9.07  (5.55, 14.8) | 0.82  (0.44, 1.51) | 0.72  (0.38, 1.35) | 23.0  (13.1, 40.2) | 0.71  (0.35, 1.45) | 0.71  (0.34, 1.49) | 0.39  (0.20, 0.76) ¶ |
| CD4+/X4+R5- | Log | 42.5  (26.1, 69.4) | 0.65  (0.36, 1.16) | 0.59  (0.33, 1.07) ‡ | 5.96  (3.43, 10.3) | 1.14  (0.58, 2.25) | 0.90  (0.44, 1.80) | 7.14  (3.83, 13.3) † |
| CD4+/X4-R5+ | Decimal | ***19.7***  ***(10.3, 29.1)*** | ***0.61***  ***(-11.1, 12.3)*** | ***-3.90***  ***(-15.9, 8.06)*** | ***47.4***  ***(36.8, 58.1)*** | ***5.72***  ***(-7.94, 19.4)*** | ***5.01***  ***(-9.05, 19.1)*** | ***-27.7***  ***(-40.3, -15.1)*** † |

**** *N = 83* Samples analyzed: N9, *n=*29; UPG, *n=*26; No gel, *n=*28; Endocervix (by curettage): *n=48,* Endometrium: *n=35*.

Significance according to Wald chi-square tests: ‡ p < 0.10; * p < 0.05; ¶ p < 0.01; † p < 0.001.
