# Supplementary material for: Unexpected Inflammatory Effects of Intravaginal Gels (Universal Placebo Gel and Nonoxynol-9) on the Upper Female Reproductive Tract: A Randomized Crossover Study
Source: PLoS One. 2015 Jul 15;10(7):e0129769. doi: 10.1371/journal.pone.0129769 (PMC4503751; doi:10.1371/journal.pone.0129769)
Supplement: S6 Table — (DOCX) [file pone.0129769.s006.docx]

**S6 Table. The complete list of differentially expressed genes in N9-exposed endometrium compared to unexposed endometrium (p<0.05, fold change ≥1.5)**

| **Gene Description** | **Gene Symbol** | **Fold Change** | **Regulation** |
| --- | --- | --- | --- |
| transcobalamin I (vitamin B12 binding protein, R binder family) | TCN1 | 2.28 | up |
| solute carrier family 1 (neuronal/epithelial high affinity glutamate transporter, system Xag), member 1 | SLC1A1 | 2.19 | up |
| prune homolog 2 (Drosophila) | PRUNE2 | 2.12 | up |
| phospholipase A2, group IIA (platelets, synovial fluid) | PLA2G2A | 2.06 | up |
| small nucleolar RNA, H/ACA box 5A | SNORA5A | 2.05 | up |
| phytanoyl-CoA 2-hydroxylase interacting protein-like \| family with sequence similarity 13, member C | PHYHIPL\|FAM13C | 2.01 | up |
| progestagen-associated endometrial protein | PAEP | 1.95 | up |
| small Cajal body-specific RNA 10 | SCARNA10 | 1.89 | up |
| gastrin | GAST | 1.89 | up |
| killer cell immunoglobulin-like receptor, two domains, long cytoplasmic tail, 3 \| killer cell immunoglobulin-like receptor, two domains, long cytoplasmic tail, 1 \| killer cell immunoglobulin-like receptor, two domains, long cytoplasmic tail, 2 \| killer cell immunoglobulin-like receptor, two domains, short cytoplasmic tail, 4 | KIR2DL3\|KIR2DL1\|KIR2DL2\|KIR2DS4 | 1.82 | up |
| GRAM domain containing 1C | GRAMD1C | 1.81 | up |
| granzyme A (granzyme 1, cytotoxic T-lymphocyte-associated serine esterase 3) | GZMA | 1.81 | up |
| small nucleolar RNA, H/ACA box 60 | SNORA60 | 1.81 | up |
| S100 calcium binding protein A1 | S100A1 | 1.80 | up |
| cathepsin W | CTSW | 1.79 | up |
| small nucleolar RNA, H/ACA box 22 | SNORA22 | 1.76 | up |
| killer cell immunoglobulin-like receptor, two domains, short cytoplasmic tail, 2 \| killer cell immunoglobulin-like receptor, two domains, long cytoplasmic tail, 2 \| killer cell immunoglobulin-like receptor, two domains, long cytoplasmic tail, 3 \| killer cell immunoglobulin-like receptor, two domains, short cytoplasmic tail, 4 \| killer cell immunoglobulin-like receptor, two domains, short cytoplasmic tail, 1 \| killer cell immunoglobulin-like receptor, two domains, long cytoplasmic tail, 1 \| killer cell immunoglobulin-like receptor, three domains, long cytoplasmic tail, 1 \| killer-cell Ig-like receptor \| killer cell immunoglobulin-like receptor, two domains, long cytoplasmic tail, 5A | KIR2DS2\|KIR2DL2\|KIR2DL3\|KIR2DS4\|KIR2DS1\|KIR2DL1\|KIR3DL1\|KIR3DP1\|KIR2DL5A | 1.75 | up |
| prune homolog 2 (Drosophila) | PRUNE2 | 1.75 | up |
| killer cell immunoglobulin-like receptor, two domains, long cytoplasmic tail, 2 \| killer cell immunoglobulin-like receptor, two domains, long cytoplasmic tail, 3 \| killer cell immunoglobulin-like receptor, two domains, long cytoplasmic tail, 1 \| killer cell immunoglobulin-like receptor, two domains, short cytoplasmic tail, 1 \| killer cell immunoglobulin-like receptor, two domains, short cytoplasmic tail, 2 | KIR2DL2\|KIR2DL3\|KIR2DL1\|KIR2DS1\|KIR2DS2 | 1.75 | up |
| aldehyde oxidase 1 | AOX1 | 1.75 | up |
| endothelin receptor type B | EDNRB | 1.73 | up |
| killer cell immunoglobulin-like receptor, two domains, short cytoplasmic tail, 4 \| killer cell immunoglobulin-like receptor, two domains, short cytoplasmic tail, 2 \| killer cell immunoglobulin-like receptor, two domains, short cytoplasmic tail, 5 \| killer cell immunoglobulin-like receptor, two domains, short cytoplasmic tail, 1 \| killer cell immunoglobulin-like receptor, three domains, short cytoplasmic tail, 1 \| killer cell immunoglobulin-like receptor, two domains, long cytoplasmic tail, 1 \| killer cell immunoglobulin-like receptor, two domains, long cytoplasmic tail, 3 \| killer cell immunoglobulin-like receptor, three domains, long cytoplasmic tail, 1 \| killer cell immunoglobulin-like receptor, two domains, long cytoplasmic tail, 5A | KIR2DS4\|KIR2DS2\|KIR2DS5\|KIR2DS1\|KIR3DS1\|KIR2DL1\|KIR2DL3\|KIR3DL1\|KIR2DL5A | 1.73 | up |
| annexin A1 | ANXA1 | 1.73 | up |
| granulysin | GNLY | 1.72 | up |
| chemokine (C motif) ligand 1 | XCL1 | 1.72 | up |
| killer cell immunoglobulin-like receptor, two domains, long cytoplasmic tail, 3 \| killer cell immunoglobulin-like receptor, two domains, long cytoplasmic tail, 2 \| killer cell immunoglobulin-like receptor, two domains, short cytoplasmic tail, 2 \| killer cell immunoglobulin-like receptor, two domains, long cytoplasmic tail, 1 \| killer cell immunoglobulin-like receptor, two domains, short cytoplasmic tail, 4 \| killer cell immunoglobulin-like receptor, two domains, long cytoplasmic tail, 5A | KIR2DL3\|KIR2DL2\|KIR2DS2\|KIR2DL1\|KIR2DS4\|KIR2DL5A | 1.69 | up |
| small nucleolar RNA, C/D box 46 | SNORD46 | 1.69 | up |
| killer cell lectin-like receptor subfamily C, member 1 | KLRC1 | 1.68 | up |
|  |  | 1.68 | up |
| killer cell immunoglobulin-like receptor, two domains, long cytoplasmic tail, 1 \| killer cell immunoglobulin-like receptor, two domains, long cytoplasmic tail, 2 \| killer cell immunoglobulin-like receptor, two domains, short cytoplasmic tail, 1 \| killer cell immunoglobulin-like receptor, two domains, long cytoplasmic tail, 3 \| killer-cell Ig-like receptor \| killer cell immunoglobulin-like receptor, two domains, short cytoplasmic tail, 4 | KIR2DL1\|KIR2DL2\|KIR2DS1\|KIR2DL3\|KIR3DP1\|KIR2DS4 | 1.67 | up |
| dynein, light chain, Tctex-type 3 | DYNLT3 | 1.66 | up |
| vault RNA 1-3 | VTRNA1-3 | 1.65 | up |
| killer cell immunoglobulin-like receptor, two domains, short cytoplasmic tail, 1 \| killer cell immunoglobulin-like receptor, two domains, short cytoplasmic tail, 4 \| killer cell immunoglobulin-like receptor, two domains, short cytoplasmic tail, 5 \| killer cell immunoglobulin-like receptor, two domains, short cytoplasmic tail, 2 \| killer cell immunoglobulin-like receptor, two domains, long cytoplasmic tail, 1 \| killer cell immunoglobulin-like receptor, two domains, long cytoplasmic tail, 3 \| killer cell immunoglobulin-like receptor, three domains, long cytoplasmic tail, 1 \| killer cell immunoglobulin-like receptor, two domains, long cytoplasmic tail, 5A | KIR2DS1\|KIR2DS4\|KIR2DS5\|KIR2DS2\|KIR2DL1\|KIR2DL3\|KIR3DL1\|KIR2DL5A | 1.65 | up |
| secreted phosphoprotein 1 | SPP1 | 1.65 | up |
| catenin (cadherin-associated protein), alpha-like 1 | CTNNAL1 | 1.64 | up |
| adhesion molecule with Ig-like domain 2 | AMIGO2 | 1.64 | up |
| metallothionein 1M | MT1M | 1.63 | up |
| ovostatin \| ovostatin 2 \| similar to hCG38149 | OVOS\|OVOS2\|LOC728715 | 1.62 | up |
| G protein-coupled receptor 160 | GPR160 | 1.62 | up |
| monoamine oxidase A | MAOA | 1.61 | up |
|  |  | 1.61 | up |
| cerebellar degeneration-related protein 1, 34kDa \| YTH domain containing 2 | CDR1\|YTHDC2 | 1.61 | up |
| microfibrillar associated protein 5 | MFAP5 | 1.60 | up |
| small Cajal body-specific RNA 7 | SCARNA7 | 1.60 | up |
|  |  | 1.59 | up |
| met proto-oncogene (hepatocyte growth factor receptor) | MET | 1.59 | up |
| ovostatin \| ovostatin 2 \| similar to hCG38149 | OVOS\|OVOS2\|LOC728715 | 1.59 | up |
| HAUS augmin-like complex, subunit 6 | HAUS6 | 1.57 | up |
| sorting nexin 10 | SNX10 | 1.56 | up |
| ribosomal modification protein rimK-like family member B | RIMKLB | 1.56 | up |
| solute carrier family 15 (oligopeptide transporter), member 1 | SLC15A1 | 1.56 | up |
| chromosome 9 open reading frame 71 | C9orf71 | 1.55 | up |
| small nucleolar RNA, H/ACA box 65 | SNORA65 | 1.55 | up |
| immediate early response 3 | IER3 | 1.55 | up |
| chromosome 19 open reading frame 33 \| Yip1 interacting factor homolog B (S. cerevisiae) | C19orf33\|YIF1B | 1.54 | up |
| killer cell immunoglobulin-like receptor, three domains, short cytoplasmic tail, 1 \| killer cell immunoglobulin-like receptor, three domains, long cytoplasmic tail, 1 \| killer cell immunoglobulin-like receptor, two domains, short cytoplasmic tail, 1 \| killer cell immunoglobulin-like receptor, two domains, long cytoplasmic tail, 3 \| killer cell immunoglobulin-like receptor, two domains, long cytoplasmic tail, 1 \| killer cell immunoglobulin-like receptor, two domains, short cytoplasmic tail, 2 \| killer cell immunoglobulin-like receptor, two domains, long cytoplasmic tail, 5A \| killer cell immunoglobulin-like receptor, two domains, short cytoplasmic tail, 4 | KIR3DS1\|KIR3DL1\|KIR2DS1\|KIR2DL3\|KIR2DL1\|KIR2DS2\|KIR2DL5A\|KIR2DS4 | 1.54 | up |
| small Cajal body-specific RNA 12 | SCARNA12 | 1.54 | up |
|  |  | 1.54 | up |
| HAUS augmin-like complex, subunit 6 | HAUS6 | 1.54 | up |
| aminoacyl tRNA synthetase complex-interacting multifunctional protein 1 \| TBC1 domain containing kinase | AIMP1\|TBCK | 1.53 | up |
| keratin 23 (histone deacetylase inducible) | KRT23 | 1.52 | up |
| solute carrier family 44, member 1 | SLC44A1 | 1.52 | up |
| eukaryotic translation initiation factor 4E family member 3 | EIF4E3 | 1.50 | up |
| neurotrophic tyrosine kinase, receptor, type 3 | NTRK3 | 1.51 | down |
| shisa homolog 6 (Xenopus laevis) | SHISA6 | 1.51 | down |
| fibronectin 1 | FN1 | 1.52 | down |
| procollagen-lysine 1, 2-oxoglutarate 5-dioxygenase 1 | PLOD1 | 1.53 | down |
| V-set and immunoglobulin domain containing 6 \| immunoglobulin heavy constant alpha 2 (A2m marker) \| immunoglobulin heavy constant gamma 1 (G1m marker) \| similar to hCG2029977 | VSIG6\|IGHA2\|IGHG1\|LOC100289944 | 1.53 | down |
| immunoglobulin kappa constant \| similar to Ig kappa chain V-I region HK102 precursor | IGKC\|LOC652493 | 1.53 | down |
|  | LOC100130876 | 1.54 | down |
| immunoglobulin kappa constant | IGKC | 1.54 | down |
| membrane-spanning 4-domains, subfamily A, member 8B | MS4A8B | 1.54 | down |
| tissue factor pathway inhibitor 2 | TFPI2 | 1.56 | down |
|  |  | 1.57 | down |
| opioid receptor, kappa 1 | OPRK1 | 1.58 | down |
| secreted frizzled-related protein 4 | SFRP4 | 1.58 | down |
|  |  | 1.60 | down |
| immunoglobulin J polypeptide, linker protein for immunoglobulin alpha and mu polypeptides | IGJ | 1.62 | down |
| kynureninase (L-kynurenine hydrolase) | KYNU | 1.62 | down |
| cytochrome P450, family 24, subfamily A, polypeptide 1 | CYP24A1 | 1.62 | down |
| lipopolysaccharide binding protein | LBP | 1.63 | down |
| immunoglobulin kappa constant | IGKC | 1.64 | down |
| cystic fibrosis transmembrane conductance regulator (ATP-binding cassette sub-family C, member 7) | CFTR | 1.68 | down |
| haptoglobin | HP | 1.68 | down |
|  |  | 1.70 | down |
| matrix metallopeptidase 26 | MMP26 | 1.71 | down |
| collagen, type I, alpha 2 | COL1A2 | 1.73 | down |
| prostaglandin-endoperoxide synthase 1 (prostaglandin G/H synthase and cyclooxygenase) | PTGS1 | 1.74 | down |
| polycystic kidney and hepatic disease 1 (autosomal recessive)-like 1 | PKHD1L1 | 1.76 | down |
| lipocalin 2 | LCN2 | 1.98 | down |
| serpin peptidase inhibitor, clade A (alpha-1 antiproteinase, antitrypsin), member 5 | SERPINA5 | 2.06 | down |
| matrix metallopeptidase 7 (matrilysin, uterine) | MMP7 | 2.11 | down |
| secretoglobin, family 1D, member 4 | SCGB1D4 | 2.15 | down |
