# Supplementary material for: Unexpected Inflammatory Effects of Intravaginal Gels (Universal Placebo Gel and Nonoxynol-9) on the Upper Female Reproductive Tract: A Randomized Crossover Study
Source: PLoS One. 2015 Jul 15;10(7):e0129769. doi: 10.1371/journal.pone.0129769 (PMC4503751; doi:10.1371/journal.pone.0129769)
Supplement: S7 Table — (DOCX) [file pone.0129769.s007.docx]

**Supplemental Table S5. The complete list of differentially expressed genes in UPG-exposed endometrium compared to unexposed endometrium (p<0.05, fold change ≥1.5)**

| **Gene description** | **Gene Symbol** | **Fold Change** | **Regulation** |
| --- | --- | --- | --- |
| gastrin | GAST | 5.75 | up |
| phospholipase A2, group IIA (platelets, synovial fluid) | PLA2G2A | 5.08 | up |
| progestagen-associated endometrial protein | PAEP | 4.07 | up |
| solute carrier family 1 (neuronal/epithelial high affinity glutamate transporter, system Xag), member 1 | SLC1A1 | 3.94 | up |
| solute carrier family 15 (oligopeptide transporter), member 1 | SLC15A1 | 3.82 | up |
| fibrinogen beta chain | FGB | 2.83 | up |
| endothelin receptor type B | EDNRB | 2.72 | up |
| hyaluronan binding protein 2 | HABP2 | 2.69 | up |
| transcobalamin I (vitamin B12 binding protein, R binder family) | TCN1 | 2.67 | up |
|  |  | 2.64 | up |
| granzyme A (granzyme 1, cytotoxic T-lymphocyte-associated serine esterase 3) | GZMA | 2.62 | up |
| secreted phosphoprotein 1 | SPP1 | 2.58 | up |
| aldehyde oxidase 1 | AOX1 | 2.54 | up |
| monoamine oxidase A | MAOA | 2.53 | up |
| cathepsin W | CTSW | 2.51 | up |
| ribosomal modification protein rimK-like family member B | RIMKLB | 2.50 | up |
| S100 calcium binding protein A1 | S100A1 | 2.48 | up |
| killer cell immunoglobulin-like receptor, two domains, long cytoplasmic tail, 3 \| killer cell immunoglobulin-like receptor, two domains, long cytoplasmic tail, 1 \| killer cell immunoglobulin-like receptor, two domains, long cytoplasmic tail, 2 \| killer cell immunoglobulin-like receptor, two domains, short cytoplasmic tail, 4 | KIR2DL3\|KIR2DL1\|KIR2DL2\|KIR2DS4 | 2.46 | up |
| granulysin | GNLY | 2.46 | up |
| chromosome 9 open reading frame 71 | C9orf71 | 2.43 | up |
| phytanoyl-CoA 2-hydroxylase interacting protein-like \| family with sequence similarity 13, member C | PHYHIPL\|FAM13C | 2.39 | up |
| solute carrier family 7 (cationic amino acid transporter, y+ system), member 2 | SLC7A2 | 2.36 | up |
| sorting nexin 10 | SNX10 | 2.31 | up |
| GRAM domain containing 1C | GRAMD1C | 2.29 | up |
| complement component 3 | C3 | 2.22 | up |
| met proto-oncogene (hepatocyte growth factor receptor) | MET | 2.16 | up |
| serpin peptidase inhibitor, clade G (C1 inhibitor), member 1 | SERPING1 | 2.16 | up |
| GLI pathogenesis-related 1 \| KRR1, small subunit (SSU) processome component, homolog (yeast) | GLIPR1\|KRR1 | 2.15 | up |
|  |  | 2.14 | up |
| UDP-N-acetyl-alpha-D-galactosamine:polypeptide N-acetylgalactosaminyltransferase 13 (GalNAc-T13) | GALNT13 | 2.12 | up |
| natural killer cell group 7 sequence | NKG7 | 2.12 | up |
| interleukin 6 signal transducer (gp130, oncostatin M receptor) | IL6ST | 2.06 | up |
| interleukin 2 receptor, beta | IL2RB | 2.06 | up |
| retinoic acid receptor responder (tazarotene induced) 3 | RARRES3 | 2.05 | up |
| AT rich interactive domain 5B (MRF1-like) | ARID5B | 2.05 | up |
| eukaryotic translation initiation factor 4E family member 3 | EIF4E3 | 2.04 | up |
| phospholipase A2, group XVI | PLA2G16 | 2.03 | up |
| cation channel, sperm-associated, beta | CATSPERB | 2.02 | up |
| acyl-CoA dehydrogenase, short/branched chain \| acyl-Coenzyme A dehydrogenase, short/branched chain | ACADSB | 2.01 | up |
| chromosome 12 open reading frame 75 | C12orf75 | 2.01 | up |
| solute carrier family 15, member 4 | SLC15A4 | 2.01 | up |
| solute carrier family 18 (vesicular monoamine), member 2 | SLC18A2 | 2.00 | up |
| nicotinamide N-methyltransferase | NNMT | 2.00 | up |
| killer cell lectin-like receptor subfamily C, member 1 | KLRC1 | 2.00 | up |
| immediate early response 3 | IER3 | 1.97 | up |
| DEAD (Asp-Glu-Ala-Asp) box polypeptide 52 | DDX52 | 1.96 | up |
| growth arrest and DNA-damage-inducible, alpha | GADD45A | 1.95 | up |
| FXYD domain containing ion transport regulator 2 | FXYD2 | 1.93 | up |
| cartilage oligomeric matrix protein | COMP | 1.93 | up |
| family with sequence similarity 134, member B | FAM134B | 1.92 | up |
| dickkopf homolog 1 (Xenopus laevis) | DKK1 | 1.92 | up |
| chromosome 10 open reading frame 10 | C10orf10 | 1.91 | up |
| stratifin | SFN | 1.91 | up |
| killer cell immunoglobulin-like receptor, two domains, short cytoplasmic tail, 4 \| killer cell immunoglobulin-like receptor, two domains, short cytoplasmic tail, 2 \| killer cell immunoglobulin-like receptor, two domains, short cytoplasmic tail, 5 \| killer cell immunoglobulin-like receptor, two domains, short cytoplasmic tail, 1 \| killer cell immunoglobulin-like receptor, three domains, short cytoplasmic tail, 1 \| killer cell immunoglobulin-like receptor, two domains, long cytoplasmic tail, 1 \| killer cell immunoglobulin-like receptor, two domains, long cytoplasmic tail, 3 \| killer cell immunoglobulin-like receptor, three domains, long cytoplasmic tail, 1 \| killer cell immunoglobulin-like receptor, two domains, long cytoplasmic tail, 5A | KIR2DS4\|KIR2DS2\|KIR2DS5\|KIR2DS1\|KIR3DS1\|KIR2DL1\|KIR2DL3\|KIR3DL1\|KIR2DL5A | 1.90 | up |
| family with sequence similarity 3, member C | FAM3C | 1.90 | up |
| S100 calcium binding protein A4 | S100A4 | 1.90 | up |
| major facilitator superfamily domain containing 4 | MFSD4 | 1.90 | up |
| SEC14 and spectrin domains 1 | SESTD1 | 1.90 | up |
| metal-regulatory transcription factor 1 | MTF1 | 1.89 | up |
| nuclear protein, transcriptional regulator, 1 | NUPR1 | 1.89 | up |
| endothelial PAS domain protein 1 | EPAS1 | 1.89 | up |
| family with sequence similarity 84, member B | FAM84B | 1.89 | up |
| calcitonin receptor-like | CALCRL | 1.89 | up |
| rhophilin, Rho GTPase binding protein 2 | RHPN2 | 1.88 | up |
| killer cell immunoglobulin-like receptor, two domains, short cytoplasmic tail, 2 \| killer cell immunoglobulin-like receptor, two domains, long cytoplasmic tail, 2 \| killer cell immunoglobulin-like receptor, two domains, long cytoplasmic tail, 3 \| killer cell immunoglobulin-like receptor, two domains, short cytoplasmic tail, 4 \| killer cell immunoglobulin-like receptor, two domains, short cytoplasmic tail, 1 \| killer cell immunoglobulin-like receptor, two domains, long cytoplasmic tail, 1 \| killer cell immunoglobulin-like receptor, three domains, long cytoplasmic tail, 1 \| killer-cell Ig-like receptor \| killer cell immunoglobulin-like receptor, two domains, long cytoplasmic tail, 5A | KIR2DS2\|KIR2DL2\|KIR2DL3\|KIR2DS4\|KIR2DS1\|KIR2DL1\|KIR3DL1\|KIR3DP1\|KIR2DL5A | 1.88 | up |
| family with sequence similarity 3, member C | FAM3C | 1.88 | up |
| versican | VCAN | 1.87 | up |
| killer cell immunoglobulin-like receptor, two domains, long cytoplasmic tail, 3 \| killer cell immunoglobulin-like receptor, two domains, long cytoplasmic tail, 2 \| killer cell immunoglobulin-like receptor, two domains, short cytoplasmic tail, 2 \| killer cell immunoglobulin-like receptor, two domains, long cytoplasmic tail, 1 \| killer cell immunoglobulin-like receptor, two domains, short cytoplasmic tail, 4 \| killer cell immunoglobulin-like receptor, two domains, long cytoplasmic tail, 5A | KIR2DL3\|KIR2DL2\|KIR2DS2\|KIR2DL1\|KIR2DS4\|KIR2DL5A | 1.87 | up |
| leucine-rich repeats and immunoglobulin-like domains 3 | LRIG3 | 1.87 | up |
| aminoacyl tRNA synthetase complex-interacting multifunctional protein 1 \| TBC1 domain containing kinase | AIMP1\|TBCK | 1.86 | up |
| metallothionein 1H | MT1H | 1.85 | up |
| guanylate cyclase 1, soluble, beta 3 | GUCY1B3 | 1.84 | up |
| killer cell immunoglobulin-like receptor, two domains, long cytoplasmic tail, 2 \| killer cell immunoglobulin-like receptor, two domains, long cytoplasmic tail, 3 \| killer cell immunoglobulin-like receptor, two domains, long cytoplasmic tail, 1 \| killer cell immunoglobulin-like receptor, two domains, short cytoplasmic tail, 1 \| killer cell immunoglobulin-like receptor, two domains, short cytoplasmic tail, 2 | KIR2DL2\|KIR2DL3\|KIR2DL1\|KIR2DS1\|KIR2DS2 | 1.84 | up |
| annexin A4 | ANXA4 | 1.84 | up |
| glutamyl aminopeptidase (aminopeptidase A) | ENPEP | 1.83 | up |
| mitogen-activated protein kinase kinase kinase 5 | MAP3K5 | 1.83 | up |
| metallothionein 1M | MT1M | 1.82 | up |
| arginase, type II \| vesicle transport through interaction with t-SNAREs homolog 1B (yeast) | ARG2\|VTI1B | 1.82 | up |
| immediate early response 3 | IER3 | 1.82 | up |
| immediate early response 3 | IER3 | 1.82 | up |
| arylsulfatase E (chondrodysplasia punctata 1) | ARSE | 1.82 | up |
| killer cell immunoglobulin-like receptor, two domains, long cytoplasmic tail, 1 \| killer cell immunoglobulin-like receptor, two domains, long cytoplasmic tail, 2 \| killer cell immunoglobulin-like receptor, two domains, short cytoplasmic tail, 1 \| killer cell immunoglobulin-like receptor, two domains, long cytoplasmic tail, 3 \| killer-cell Ig-like receptor \| killer cell immunoglobulin-like receptor, two domains, short cytoplasmic tail, 4 | KIR2DL1\|KIR2DL2\|KIR2DS1\|KIR2DL3\|KIR3DP1\|KIR2DS4 | 1.82 | up |
| granzyme B (granzyme 2, cytotoxic T-lymphocyte-associated serine esterase 1) | GZMB | 1.82 | up |
|  | CD68 | 1.82 | up |
| complement component 1, r subcomponent | C1R | 1.81 | up |
| beta-1,4-N-acetyl-galactosaminyl transferase 3 | B4GALNT3 | 1.80 | up |
| arylsulfatase B | ARSB | 1.80 | up |
| dynein, light chain, Tctex-type 3 | DYNLT3 | 1.78 | up |
| vault RNA 1-3 | VTRNA1-3 | 1.78 | up |
| filamin A interacting protein 1 | FILIP1 | 1.78 | up |
| tubulin, alpha 4a | TUBA4A | 1.78 | up |
| ATPase, H+ transporting, lysosomal 70kDa, V1 subunit A | ATP6V1A | 1.77 | up |
| ATP-binding cassette, sub-family C (CFTR/MRP), member 3 | ABCC3 | 1.77 | up |
| HNF1 homeobox B | HNF1B | 1.75 | up |
| anterior gradient homolog 2 (Xenopus laevis) | AGR2 | 1.75 | up |
| solute carrier family 44, member 1 | SLC44A1 | 1.75 | up |
| ephrin-A1 | EFNA1 | 1.74 | up |
| cytidine deaminase | CDA | 1.74 | up |
| chromosome 12 open reading frame 27 | C12orf27 | 1.74 | up |
| apolipoprotein C-I | APOC1 | 1.73 | up |
| thrombospondin, type I, domain containing 7A | THSD7A | 1.73 | up |
| family with sequence similarity 60, member A | FAM60A | 1.73 | up |
| angiopoietin-like 1 | ANGPTL1 | 1.72 | up |
| inhibitor of DNA binding 4, dominant negative helix-loop-helix protein | ID4 | 1.72 | up |
| Rho GTPase activating protein 29 | ARHGAP29 | 1.72 | up |
| F-box protein 32 | FBXO32 | 1.72 | up |
| anthrax toxin receptor 2 | ANTXR2 | 1.71 | up |
| guanylate binding protein 2, interferon-inducible | GBP2 | 1.71 | up |
| killer cell immunoglobulin-like receptor, two domains, long cytoplasmic tail, 4 \| killer cell immunoglobulin-like receptor, two domains, long cytoplasmic tail, 3 \| killer cell immunoglobulin-like receptor, two domains, long cytoplasmic tail, 5A | KIR2DL4\|KIR2DL3\|KIR2DL5A | 1.71 | up |
| hepatitis A virus cellular receptor 1 | HAVCR1 | 1.71 | up |
| complement component 1, s subcomponent | C1S | 1.70 | up |
| histidine ammonia-lyase | HAL | 1.70 | up |
| chemokine (C-X-C motif) ligand 13 | CXCL13 | 1.70 | up |
| mal, T-cell differentiation protein | MAL | 1.69 | up |
| FXYD domain containing ion transport regulator 2 | FXYD2 | 1.69 | up |
| protein tyrosine phosphatase, receptor type, C | PTPRC | 1.69 | up |
| insulin-like growth factor 2 (somatomedin A) \| INS-IGF2 readthrough transcript | IGF2\|INS-IGF2 | 1.68 | up |
| coactosin-like 1 (Dictyostelium) | COTL1 | 1.67 | up |
|  |  | 1.67 | up |
| G protein-coupled receptor 64 | GPR64 | 1.67 | up |
| interleukin 15 | IL15 | 1.66 | up |
| claudin 4 | CLDN4 | 1.66 | up |
| retinol dehydrogenase 10 (all-trans) | RDH10 | 1.66 | up |
| indoleamine 2,3-dioxygenase 2 | IDO2 | 1.66 | up |
| myocardin | MYOCD | 1.66 | up |
| clusterin | CLU | 1.66 | up |
| tandem C2 domains, nuclear | TC2N | 1.66 | up |
| huntingtin-associated protein 1 | HAP1 | 1.66 | up |
| catenin (cadherin-associated protein), alpha-like 1 | CTNNAL1 | 1.65 | up |
| TIMP metallopeptidase inhibitor 3 | TIMP3 | 1.65 | up |
| ovostatin \| ovostatin 2 \| similar to hCG38149 | OVOS\|OVOS2\|LOC728715 | 1.65 | up |
| chromodomain protein, Y-like 2 | CDYL2 | 1.64 | up |
| killer cell lectin-like receptor subfamily C, member 4 | KLRC4 | 1.64 | up |
| killer cell immunoglobulin-like receptor, two domains, long cytoplasmic tail, 4 \| killer cell immunoglobulin-like receptor, two domains, long cytoplasmic tail, 3 \| killer cell immunoglobulin-like receptor, two domains, long cytoplasmic tail, 5A | KIR2DL4\|KIR2DL3\|KIR2DL5A | 1.64 | up |
| killer cell immunoglobulin-like receptor, two domains, short cytoplasmic tail, 1 \| killer cell immunoglobulin-like receptor, two domains, short cytoplasmic tail, 4 \| killer cell immunoglobulin-like receptor, two domains, short cytoplasmic tail, 5 \| killer cell immunoglobulin-like receptor, two domains, short cytoplasmic tail, 2 \| killer cell immunoglobulin-like receptor, two domains, long cytoplasmic tail, 1 \| killer cell immunoglobulin-like receptor, two domains, long cytoplasmic tail, 3 \| killer cell immunoglobulin-like receptor, three domains, long cytoplasmic tail, 1 \| killer cell immunoglobulin-like receptor, two domains, long cytoplasmic tail, 5A | KIR2DS1\|KIR2DS4\|KIR2DS5\|KIR2DS2\|KIR2DL1\|KIR2DL3\|KIR3DL1\|KIR2DL5A | 1.64 | up |
| solute carrier family 3 (cystine, dibasic and neutral amino acid transporters, activator of cystine, dibasic and neutral amino acid transport), member 1 \| prolyl endopeptidase-like | SLC3A1\|PREPL | 1.63 | up |
| killer cell lectin-like receptor subfamily K, member 1 \| killer cell lectin-like receptor subfamily C, member 4 | KLRK1\|KLRC4 | 1.63 | up |
| fibulin 5 | FBLN5 | 1.63 | up |
| heat shock protein, alpha-crystallin-related, B6 | HSPB6 | 1.63 | up |
| ovostatin \| ovostatin 2 \| similar to hCG38149 | OVOS\|OVOS2\|LOC728715 | 1.62 | up |
| DEP domain containing 1B | DEPDC1B | 1.62 | up |
| aquaporin 3 (Gill blood group) | AQP3 | 1.62 | up |
| fibroblast growth factor 10 | FGF10 | 1.61 | up |
| transient receptor potential cation channel, subfamily C, member 6 | TRPC6 | 1.61 | up |
| lysozyme \| lysozyme (renal amyloidosis) | LYZ | 1.60 | up |
| dedicator of cytokinesis 10 | DOCK10 | 1.60 | up |
| tetraspanin 12 | TSPAN12 | 1.59 | up |
| adhesion molecule with Ig-like domain 2 | AMIGO2 | 1.58 | up |
| chromosome 1 open reading frame 130 | C1orf130 | 1.58 | up |
| 1-acylglycerol-3-phosphate O-acyltransferase 5 (lysophosphatidic acid acyltransferase, epsilon) | AGPAT5 | 1.58 | up |
| gamma-aminobutyric acid (GABA) A receptor, alpha 2 | GABRA2 | 1.58 | up |
| carbonic anhydrase XII | CA12 | 1.58 | up |
| Fc fragment of IgE, high affinity I, receptor for; gamma polypeptide | FCER1G | 1.58 | up |
| H19, imprinted maternally expressed transcript (non-protein coding) \| microRNA 675 | H19\|MIR675 | 1.57 | up |
| saccharopine dehydrogenase (putative) | SCCPDH | 1.57 | up |
| ephrin-A5 | EFNA5 | 1.57 | up |
| mitochondrial ribosomal protein S2 | MRPS2 | 1.57 | up |
|  | FYB | 1.57 | up |
| laminin, alpha 4 | LAMA4 | 1.57 | up |
| chemokine (C-C motif) ligand 21 | CCL21 | 1.56 | up |
| solute carrier family 8 (sodium/calcium exchanger), member 1 | SLC8A1 | 1.56 | up |
| G protein-coupled receptor, family C, group 5, member B | GPRC5B | 1.56 | up |
| toll-like receptor 4 | TLR4 | 1.55 | up |
| RNA, U2 small nuclear 1 \| RNA, U2 small nuclear 2 \| WD repeat domain 74 | RNU2-1\|RNU2-2\|WDR74 | 1.55 | up |
| RNA, U2 small nuclear 1 \| RNA, U2 small nuclear 2 \| WD repeat domain 74 | RNU2-1\|RNU2-2\|WDR74 | 1.55 | up |
| RNA, U2 small nuclear 1 \| RNA, U2 small nuclear 2 \| WD repeat domain 74 | RNU2-1\|RNU2-2\|WDR74 | 1.55 | up |
| RNA, U2 small nuclear 1 \| RNA, U2 small nuclear 2 \| WD repeat domain 74 | RNU2-1\|RNU2-2\|WDR74 | 1.55 | up |
| RNA, U2 small nuclear 1 \| RNA, U2 small nuclear 2 \| WD repeat domain 74 | RNU2-1\|RNU2-2\|WDR74 | 1.55 | up |
| RNA, U2 small nuclear 1 \| RNA, U2 small nuclear 2 \| WD repeat domain 74 | RNU2-1\|RNU2-2\|WDR74 | 1.55 | up |
| RNA, U2 small nuclear 1 \| RNA, U2 small nuclear 2 \| WD repeat domain 74 | RNU2-1\|RNU2-2\|WDR74 | 1.55 | up |
| RNA, U2 small nuclear 1 \| RNA, U2 small nuclear 2 \| WD repeat domain 74 | RNU2-1\|RNU2-2\|WDR74 | 1.55 | up |
| RNA, U2 small nuclear 1 \| RNA, U2 small nuclear 2 \| WD repeat domain 74 | RNU2-1\|RNU2-2\|WDR74 | 1.55 | up |
| elongation factor, RNA polymerase II, 2 | ELL2 | 1.55 | up |
| WD repeat domain 72 | WDR72 | 1.54 | up |
| leiomodin 1 (smooth muscle) | LMOD1 | 1.54 | up |
| cell division cycle 7 homolog (S. cerevisiae) | CDC7 | 1.54 | up |
| myosin, light chain 9, regulatory | MYL9 | 1.54 | up |
| laminin, beta 3 | LAMB3 | 1.54 | up |
| LIM and cysteine-rich domains 1 | LMCD1 | 1.54 | up |
| chemokine (C-X-C motif) receptor 4 | CXCR4 | 1.54 | up |
| C2 calcium-dependent domain containing 4A \| family with sequence similarity 148, member A | C2CD4A\|FAM148A | 1.53 | up |
| RNA, U2 small nuclear 1 \| RNA, U2 small nuclear 2 \| WD repeat domain 74 | RNU2-1\|RNU2-2\|WDR74 | 1.53 | up |
| protein C receptor, endothelial \| protein C receptor, endothelial (EPCR) | PROCR | 1.53 | up |
| RNA, U2 small nuclear 1 \| RNA, U2 small nuclear 2 \| WD repeat domain 74 | RNU2-1\|RNU2-2\|WDR74 | 1.53 | up |
| interleukin 15 receptor, alpha | IL15RA | 1.53 | up |
| amiloride binding protein 1 (amine oxidase (copper-containing)) | ABP1 | 1.53 | up |
|  |  | 1.53 | up |
| indoleamine 2,3-dioxygenase 1 | IDO1 | 1.53 | up |
| transmembrane protein 37 | TMEM37 | 1.53 | up |
| killer cell immunoglobulin-like receptor, three domains, short cytoplasmic tail, 1 \| killer cell immunoglobulin-like receptor, three domains, long cytoplasmic tail, 1 \| killer cell immunoglobulin-like receptor, two domains, short cytoplasmic tail, 1 \| killer cell immunoglobulin-like receptor, two domains, long cytoplasmic tail, 3 \| killer cell immunoglobulin-like receptor, two domains, long cytoplasmic tail, 1 \| killer cell immunoglobulin-like receptor, two domains, short cytoplasmic tail, 2 \| killer cell immunoglobulin-like receptor, two domains, long cytoplasmic tail, 5A \| killer cell immunoglobulin-like receptor, two domains, short cytoplasmic tail, 4 | KIR3DS1\|KIR3DL1\|KIR2DS1\|KIR2DL3\|KIR2DL1\|KIR2DS2\|KIR2DL5A\|KIR2DS4 | 1.53 | up |
| perforin 1 (pore forming protein) | PRF1 | 1.53 | up |
| transmembrane protein 92 | TMEM92 | 1.52 | up |
| endoplasmic reticulum to nucleus signaling 1 | ERN1 | 1.52 | up |
| transient receptor potential cation channel, subfamily C, member 4 | TRPC4 | 1.52 | up |
| argininosuccinate synthase 1 \| argininosuccinate synthetase 1 | ASS1 | 1.52 | up |
| inhibitor of DNA binding 2, dominant negative helix-loop-helix protein | ID2 | 1.52 | up |
| claudin 1 | CLDN1 | 1.51 | up |
| C-type lectin domain family 4, member E | CLEC4E | 1.51 | up |
| thrombomodulin | THBD | 1.51 | up |
| histamine N-methyltransferase | HNMT | 1.51 | up |
| branched chain amino-acid transaminase 1, cytosolic \| branched chain aminotransferase 1, cytosolic | BCAT1 | 1.51 | up |
| sorting nexin 29 | SNX29 | 1.51 | up |
|  | KIAA0040 | 1.51 | up |
| galactosidase, beta 1-like 2 | GLB1L2 | 1.50 | up |
| metallothionein 1L (gene/pseudogene) | MT1L | 1.50 | up |
| hypothetical LOC339524 \| heparan sulfate 2-O-sulfotransferase 1 | LOC339524\|HS2ST1 | 1.50 | up |
| fibrinogen alpha chain | FGA | 1.50 | up |
| scavenger receptor class A, member 5 (putative) | SCARA5 | 1.50 | up |
| apolipoprotein E \| high mobility group AT-hook 1 | APOE\|HMGA1 | 1.50 | up |
| retinoblastoma binding protein 8 | RBBP8 | 1.50 | up |
| small nucleolar RNA, C/D box 46 | SNORD46 | 1.50 | up |
| suppressor of fused homolog (Drosophila) | SUFU | 1.50 | down |
| anoctamin 10 | ANO10 | 1.50 | down |
| WD repeat domain 77 | WDR77 | 1.50 | down |
|  |  | 1.50 | down |
| Ras association (RalGDS/AF-6) domain family member 4 | RASSF4 | 1.50 | down |
| immunoglobulin heavy constant mu \| similar to Ig heavy chain V-III region VH26 precursor | IGHM\|LOC652494 | 1.50 | down |
| hairy/enhancer-of-split related with YRPW motif-like | HEYL | 1.50 | down |
| phospholipase C, beta 1 (phosphoinositide-specific) | PLCB1 | 1.50 | down |
| transducin-like enhancer of split 1 (E(sp1) homolog, Drosophila) | TLE1 | 1.50 | down |
| kynureninase (L-kynurenine hydrolase) | KYNU | 1.51 | down |
| immunoglobulin kappa constant \| similar to Ig kappa chain V-I region HK102 precursor | IGKC\|LOC652493 | 1.51 | down |
| ubiquitin specific peptidase 53 | USP53 | 1.51 | down |
| 5'-nucleotidase domain containing 3 | NT5DC3 | 1.51 | down |
| DENN/MADD domain containing 2C | DENND2C | 1.51 | down |
| zinc finger protein 652 | ZNF652 | 1.51 | down |
| uridine-cytidine kinase 2 | UCK2 | 1.51 | down |
| pyrophosphatase (inorganic) 1 | PPA1 | 1.51 | down |
| immunoglobulin kappa constant | IGKC | 1.52 | down |
| multiple EGF-like-domains 10 | MEGF10 | 1.52 | down |
| ST3 beta-galactoside alpha-2,3-sialyltransferase 6 | ST3GAL6 | 1.52 | down |
| hairy/enhancer-of-split related with YRPW motif 1 | HEY1 | 1.52 | down |
| nephroblastoma overexpressed gene | NOV | 1.52 | down |
| spermidine/spermine N1-acetyltransferase 1 | SAT1 | 1.52 | down |
| zinc finger protein 750 | ZNF750 | 1.52 | down |
| chromosome 15 open reading frame 48 \| microRNA 147b | C15orf48\|MIR147B | 1.52 | down |
| serum amyloid A1 | SAA1 | 1.53 | down |
| anaphase promoting complex subunit 4 | ANAPC4 | 1.53 | down |
| mitochondrial carrier protein-like | LOC153328 | 1.53 | down |
| folate receptor 1 (adult) | FOLR1 | 1.54 | down |
| solute carrier family 25, member 38 | SLC25A38 | 1.54 | down |
| melanoma antigen \| hypothetical gene supported by AK096952; AK126241; BC068588 \| hypothetical protein LOC100132288 \| similar to tektin 4 \| MAFF interacting protein \| hypothetical protein LOC100233156 | LOC51152\|FLJ44253\|LOC100132288\|LOC727768\|MAFIP\|LOC100233156 | 1.54 | down |
| fibronectin 1 | FN1 | 1.54 | down |
| M-phase phosphoprotein 10 (U3 small nucleolar ribonucleoprotein) | MPHOSPH10 | 1.54 | down |
| ATPase, H+ transporting V0 subunit e2 | ATP6V0E2 | 1.55 | down |
| procollagen-lysine 1, 2-oxoglutarate 5-dioxygenase 1 | PLOD1 | 1.55 | down |
| fyn-related kinase | FRK | 1.55 | down |
| gap junction protein, beta 6, 30kDa | GJB6 | 1.56 | down |
| hydroxysteroid (17-beta) dehydrogenase 2 | HSD17B2 | 1.56 | down |
| hypothetical protein LOC100132288 \| MAFF interacting protein | LOC100132288\|MAFIP | 1.57 | down |
| zinc finger protein 814 \| zinc finger protein 552 \| zinc finger protein 587 \| similar to zinc finger protein 587 | ZNF814\|ZNF552\|ZNF587\|LOC100288322 | 1.57 | down |
| cellular retinoic acid binding protein 2 | CRABP2 | 1.57 | down |
| PHD finger protein 8 | PHF8 | 1.57 | down |
| V-set and immunoglobulin domain containing 6 \| immunoglobulin heavy constant alpha 2 (A2m marker) \| immunoglobulin heavy constant gamma 1 (G1m marker) \| similar to hCG2029977 | VSIG6\|IGHA2\|IGHG1\|LOC100289944 | 1.57 | down |
| UDP glucuronosyltransferase 2 family, polypeptide B7 | UGT2B7 | 1.57 | down |
| non-protein coding RNA 116 | NCRNA00116 | 1.57 | down |
| c-mer proto-oncogene tyrosine kinase | MERTK | 1.57 | down |
| chromosome 9 open reading frame 31 | C9orf31 | 1.57 | down |
| hypoxanthine phosphoribosyltransferase 1 | HPRT1 | 1.58 | down |
| IKAROS family zinc finger 2 (Helios) | IKZF2 | 1.58 | down |
| UDP glucuronosyltransferase 2 family, polypeptide B7 | UGT2B7 | 1.58 | down |
| RAD54 homolog B (S. cerevisiae) | RAD54B | 1.58 | down |
| serum amyloid A2 | SAA2 | 1.58 | down |
|  | LOC100190938 | 1.58 | down |
| transmembrane 7 superfamily member 3 | TM7SF3 | 1.59 | down |
| protein kinase, X-linked | PRKX | 1.59 | down |
| ankylosis, progressive homolog (mouse) | ANKH | 1.59 | down |
| mitochondrial ribosomal protein L3 | MRPL3 | 1.59 | down |
| RAN binding protein 17 | RANBP17 | 1.59 | down |
| tetraspanin 13 | TSPAN13 | 1.59 | down |
| protein phosphatase, Mg2+/Mn2+ dependent, 1H \| protein phosphatase 1H (PP2C domain containing) | PPM1H | 1.60 | down |
| transmembrane protein 101 | TMEM101 | 1.60 | down |
| chromosome 1 open reading frame 113 \| family with sequence similarity 176, member B | C1orf113\|FAM176B | 1.60 | down |
|  | RAB25 | 1.61 | down |
| solute carrier family 25 (mitochondrial carrier; citrate transporter), member 1 | SLC25A1 | 1.61 | down |
| seven in absentia homolog 3 (Drosophila) | SIAH3 | 1.61 | down |
| immunoglobulin kappa locus \| immunoglobulin kappa constant | IGK@\|IGKC | 1.62 | down |
| SH3 domain containing ring finger 2 | SH3RF2 | 1.62 | down |
| cytochrome P450, family 24, subfamily A, polypeptide 1 | CYP24A1 | 1.62 | down |
|  |  | 1.62 | down |
| cystatin SN | CST1 | 1.62 | down |
| stathmin 1 | STMN1 | 1.62 | down |
| transcription elongation factor A (SII), 3 | TCEA3 | 1.63 | down |
| matrilin 2 | MATN2 | 1.63 | down |
| hemoglobin, alpha 1 \| hemoglobin, alpha 2 | HBA1\|HBA2 | 1.63 | down |
| hemoglobin, alpha 2 \| hemoglobin, alpha 1 | HBA2\|HBA1 | 1.63 | down |
| immunoglobulin kappa constant | IGKC | 1.64 | down |
| heat shock 70kDa protein 2 | HSPA2 | 1.64 | down |
| retinol binding protein 7, cellular | RBP7 | 1.64 | down |
| chromosome 5 open reading frame 13 | C5orf13 | 1.65 | down |
| thioesterase superfamily member 4 | THEM4 | 1.65 | down |
| BCL2-associated athanogene 5 | BAG5 | 1.65 | down |
| syntaxin 18 | STX18 | 1.65 | down |
| transmembrane emp24 protein transport domain containing 6 | TMED6 | 1.65 | down |
| SMAD family member 9 | SMAD9 | 1.66 | down |
| MOCO sulphurase C-terminal domain containing 1 | MOSC1 | 1.66 | down |
| cordon-bleu homolog (mouse) | COBL | 1.66 | down |
| POC1 centriolar protein homolog B (Chlamydomonas) \| WD repeat domain 51B \| UDP-N-acetyl-alpha-D-galactosamine:polypeptide N-acetylgalactosaminyltransferase 4 (GalNAc-T4) | POC1B\|WDR51B\|GALNT4 | 1.66 | down |
| transmembrane protein 132C | TMEM132C | 1.66 | down |
| endothelin 3 | EDN3 | 1.67 | down |
| Ras association (RalGDS/AF-6) domain family member 2 | RASSF2 | 1.67 | down |
|  |  | 1.68 | down |
| family with sequence similarity 169, member A | FAM169A | 1.68 | down |
|  |  | 1.68 | down |
| frizzled homolog 5 (Drosophila) | FZD5 | 1.68 | down |
| sorting nexin family member 30 | SNX30 | 1.69 | down |
| matrix metallopeptidase 7 (matrilysin, uterine) | MMP7 | 1.70 | down |
|  | KIAA1324 | 1.70 | down |
| gelsolin \| gelsolin (amyloidosis, Finnish type) | GSN | 1.71 | down |
| nucleoporin 153kDa | NUP153 | 1.71 | down |
| estrogen receptor 1 | ESR1 | 1.71 | down |
| paired box 2 | PAX2 | 1.71 | down |
| UDP-N-acetyl-alpha-D-galactosamine:polypeptide N-acetylgalactosaminyltransferase 4 (GalNAc-T4) \| POC1 centriolar protein homolog B (Chlamydomonas) \| WD repeat domain 51B | GALNT4\|POC1B\|WDR51B | 1.71 | down |
| nuclear factor I/B | NFIB | 1.72 | down |
| gremlin 2, cysteine knot superfamily, homolog (Xenopus laevis) | GREM2 | 1.72 | down |
| pyruvate dehydrogenase kinase, isozyme 4 | PDK4 | 1.72 | down |
| glycoprotein 2 (zymogen granule membrane) | GP2 | 1.72 | down |
| troponin C type 1 (slow) | TNNC1 | 1.72 | down |
| plakophilin 2 | PKP2 | 1.73 | down |
| prostaglandin-endoperoxide synthase 2 (prostaglandin G/H synthase and cyclooxygenase) | PTGS2 | 1.73 | down |
| phosphatidylinositol-4-phosphate 5-kinase, type I, beta | PIP5K1B | 1.73 | down |
| solute carrier family 4, sodium bicarbonate cotransporter, member 7 | SLC4A7 | 1.73 | down |
| peptidylglycine alpha-amidating monooxygenase | PAM | 1.73 | down |
| dehydrogenase/reductase (SDR family) member 7 | DHRS7 | 1.74 | down |
| potassium voltage-gated channel, subfamily G, member 1 | KCNG1 | 1.74 | down |
| synaptotagmin II | SYT2 | 1.74 | down |
| melanoma antigen family D, 1 | MAGED1 | 1.74 | down |
| homer homolog 2 (Drosophila) | HOMER2 | 1.75 | down |
| prostate androgen-regulated transcript 1 (non-protein coding) \| phosphodiesterase 4D, cAMP-specific (phosphodiesterase E3 dunce homolog, Drosophila) | PART1\|PDE4D | 1.76 | down |
| gap junction protein, beta 2, 26kDa | GJB2 | 1.76 | down |
| neurotrophic tyrosine kinase, receptor, type 3 | NTRK3 | 1.77 | down |
| receptor (G protein-coupled) activity modifying protein 2 | RAMP2 | 1.78 | down |
| immunoglobulin J polypeptide, linker protein for immunoglobulin alpha and mu polypeptides | IGJ | 1.78 | down |
| trafficking protein, kinesin binding 1 | TRAK1 | 1.78 | down |
| chromosome 14 open reading frame 153 \| kinesin light chain 1 | C14orf153\|KLC1 | 1.78 | down |
| branched chain keto acid dehydrogenase E1, beta polypeptide | BCKDHB | 1.79 | down |
| guanine nucleotide binding protein (G protein), gamma 11 | GNG11 | 1.80 | down |
| RasGEF domain family, member 1B | RASGEF1B | 1.80 | down |
| Rho GTPase activating protein 26 | ARHGAP26 | 1.80 | down |
| homogentisate 1,2-dioxygenase \| homogentisate 1,2-dioxygenase (homogentisate oxidase) | HGD | 1.80 | down |
| discs, large homolog 5 (Drosophila) | DLG5 | 1.80 | down |
| homogentisate 1,2-dioxygenase \| homogentisate 1,2-dioxygenase (homogentisate oxidase) | HGD | 1.81 | down |
| SAM pointed domain containing ets transcription factor | SPDEF | 1.82 | down |
| hairy/enhancer-of-split related with YRPW motif 2 | HEY2 | 1.82 | down |
| solute carrier family 39 (zinc transporter), member 14 | SLC39A14 | 1.83 | down |
| CDC14 cell division cycle 14 homolog A (S. cerevisiae) | CDC14A | 1.83 | down |
| solute carrier family 7 (cationic amino acid transporter, y+ system), member 4 | SLC7A4 | 1.87 | down |
| carbonic anhydrase II | CA2 | 1.88 | down |
| hemoglobin, beta | HBB | 1.88 | down |
| transmembrane 4 L six family member 4 | TM4SF4 | 1.88 | down |
| proline rich, lacrimal 1 | PROL1 | 1.88 | down |
| cytochrome b5 type A (microsomal) | CYB5A | 1.89 | down |
| OCIA domain containing 2 | OCIAD2 | 1.89 | down |
| neural precursor cell expressed, developmentally down-regulated 9 | NEDD9 | 1.89 | down |
| zinc finger protein 589 | ZNF589 | 1.90 | down |
| phospholipase C, eta 1 | PLCH1 | 1.91 | down |
| guanosine monophosphate reductase | GMPR | 1.93 | down |
| elongation protein 3 homolog (S. cerevisiae) | ELP3 | 1.93 | down |
| WAP four-disulfide core domain 2 | WFDC2 | 1.95 | down |
| Fraser syndrome 1 | FRAS1 | 1.98 | down |
| hydroxysteroid (11-beta) dehydrogenase 2 | HSD11B2 | 1.98 | down |
| serpin peptidase inhibitor, clade B (ovalbumin), member 9 | SERPINB9 | 1.99 | down |
| ATPase, Na+/K+ transporting, beta 1 polypeptide \| non-metastatic cells 7, protein expressed in (nucleoside-diphosphate kinase) | ATP1B1\|NME7 | 1.99 | down |
| solute carrier family 43, member 1 | SLC43A1 | 1.99 | down |
| ankyrin 3, node of Ranvier (ankyrin G) | ANK3 | 2.01 | down |
| peroxisomal D3,D2-enoyl-CoA isomerase \| chromosome 6 open reading frame 201 | PECI\|C6orf201 | 2.03 | down |
| secreted frizzled-related protein 4 | SFRP4 | 2.03 | down |
| phosphatidylethanolamine-binding protein 4 | PEBP4 | 2.04 | down |
| asparaginase like 1 | ASRGL1 | 2.05 | down |
| cytochrome P450, family 2, subfamily J, polypeptide 2 | CYP2J2 | 2.06 | down |
| transmembrane protein 144 | TMEM144 | 2.07 | down |
| major histocompatibility complex, class II, DO beta \| transporter 2, ATP-binding cassette, sub-family B (MDR/TAP) | HLA-DOB\|TAP2 | 2.08 | down |
| metallophosphoesterase domain containing 2 | MPPED2 | 2.09 | down |
| ADAM metallopeptidase with thrombospondin type 1 motif, 8 | ADAMTS8 | 2.10 | down |
| isocitrate dehydrogenase 1 (NADP+), soluble | IDH1 | 2.11 | down |
| major histocompatibility complex, class II, DO beta \| transporter 2, ATP-binding cassette, sub-family B (MDR/TAP) | HLA-DOB\|TAP2 | 2.13 | down |
| creatine kinase, brain | CKB | 2.15 | down |
| collagen, type I, alpha 2 | COL1A2 | 2.16 | down |
| ectonucleotide pyrophosphatase/phosphodiesterase 3 | ENPP3 | 2.19 | down |
| cytochrome P450, family 26, subfamily A, polypeptide 1 | CYP26A1 | 2.20 | down |
| SLAIN motif family, member 1 | SLAIN1 | 2.21 | down |
| leucine rich repeat containing 1 | LRRC1 | 2.29 | down |
| glycoprotein, alpha-galactosyltransferase 1 pseudogene | GGTA1 | 2.29 | down |
| opioid receptor, kappa 1 | OPRK1 | 2.29 | down |
| FXYD domain containing ion transport regulator 4 | FXYD4 | 2.33 | down |
| transient receptor potential cation channel, subfamily M, member 6 | TRPM6 | 2.38 | down |
| tissue factor pathway inhibitor 2 | TFPI2 | 2.54 | down |
| catenin (cadherin-associated protein), alpha 2 | CTNNA2 | 2.56 | down |
| NDRG family member 2 | NDRG2 | 2.57 | down |
| cAMP responsive element binding protein 3-like 1 | CREB3L1 | 2.65 | down |
| calpain 6 | CAPN6 | 2.76 | down |
| xanthine dehydrogenase | XDH | 2.78 | down |
| alkaline phosphatase, liver/bone/kidney | ALPL | 2.88 | down |
| secretoglobin, family 1D, member 4 | SCGB1D4 | 3.06 | down |
| serpin peptidase inhibitor, clade A (alpha-1 antiproteinase, antitrypsin), member 5 | SERPINA5 | 3.28 | down |
| matrix metallopeptidase 26 | MMP26 | 4.82 | down |
| polycystic kidney and hepatic disease 1 (autosomal recessive)-like 1 | PKHD1L1 | 5.27 | down |
